# ﻿Two new species of the *Cnemaspisgalaxia* complex (Squamata, Gekkonidae) from the eastern slopes of the southern Western Ghats

**DOI:** 10.3897/zookeys.1196.117947

**Published:** 2024-03-27

**Authors:** Akshay Khandekar, Tejas Thackeray, Ishan Agarwal

**Affiliations:** 1 Thackeray Wildlife Foundation, Mumbai, 400051, India Thackeray Wildlife Foundation Mumbai India; 2 Department of Zoology, Shivaji University, Kolhapur, 416004, India Shivaji University Kolhapur India

**Keywords:** Asia, biodiversity hotspot, dwarf geckos, integrative taxonomy, phylogeny, species complex

## Abstract

Two new species allied to *Cnemaspisgalaxia* are described from the eastern slopes of the south Western Ghats, Tamil Nadu, India. Both new species are members of the *ornata* subclade within the *beddomei* clade. The two new species can be easily distinguished from all other members of the *beddomei* clade and each other by a combination of nonoverlapping morphological characters such as small body size, distinct colouration of both sexes, the number of dorsal tubercles around the body, the number or arrangement of paravertebral tubercles, the number of midventral scales across the belly and longitudinal ventral scales from mental to cloaca, besides uncorrected pairwise ND2 and 16S sequence divergence of ≥ 7.4% and ≥ 2.7%. The two new species are distributed from low elevation, deciduous forests of Srivilliputhur, and add to the five previously known endemic vertebrates from Srivilliputhur-Megamalai Tiger Reserve.

## ﻿Introduction

The *beddomei* clade is the only clade of South Asian *Cnemaspis* restricted to the southern Western Ghats (Cyriac et al. 2018; Sayyed et al. 2019, 2023a, b; Pal et al. 2021). All members of the *beddomei* clade are brightly coloured in life and the first two species were described more than 150 years ago (Beddome 1870; Theobald 1876; Cyriac et al. 2018; Sayyed et al. 2019, 2023a, b; Pal et al. 2021; Khandekar et al. 2022). However, this clade was only recently sampled and recognised in a molecular phylogeny of South Asian *Cnemaspis* (Pal et al. 2021). As currently understood, the *beddomei* clade began diversifying in the Eocene and includes 16 described species, distributed from scrub to evergreen forests on the eastern and western slopes of the Western Ghats south of the Palghat Gap (Cyriac et al. 2018; Sayyed et al. 2019, 2023a, b; Pal et al. 2021; Khandekar et al. 2022). The clade is made up of three deeply divergent, well-supported subclades — the *anamudiensis* subclade with three species, *C.anamudiensis* Cyriac, Johny, Umesh & Palot, 2018, *C.nimbus* Pal, Mirza, Dsouza & Shanker, 2021, and *C.wallaceii* Pal, Mirza, Dsouza & Shanker, 2021; the *beddomei* subclade with four species, *C.beddomei* (Beddome, 1870), *C.maculicollis* Cyriac, Johny, Umesh & Palot, 2018, *C.smaug* Pal, Mirza, Dsouza & Shanker, 2021, and *C.rubraoculus* Pal, Mirza, Dsouza & Shanker, 2021; and the *ornata* subclade with nine species, *C.ornata* (Beddome, 1870), *C.aaronbaueri* Sayyed, Grismer, Campbell & Dileepkumar, 2019, *C.azhagu* Khandekar, Thackeray & Agarwal, 2022, *C.galaxia* Pal, Mirza, Dsouza & Shanker, 2021, *C.nairi* Inger, Marx & Koshy, 1984, *C.nigriventris* Pal, Mirza, Dsouza & Shanker, 2021, *C.regalis* Pal, Mirza, Dsouza & Shanker, 2021, *C.rashidi* Sayyed et al., 2023, and *C.sundara* Sayyed et al., 2023 (Cyriac et al. 2018; Sayyed et al. 2019, 2023a, b; Pal et al. 2021; Khandekar et al. 2022).

**Figure 1.**
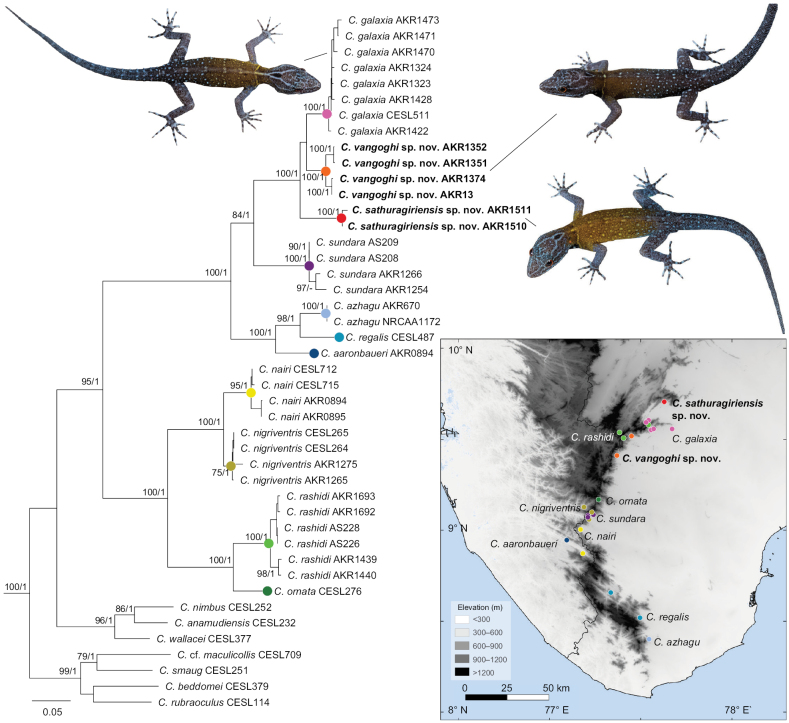
Maximum likelihood phylogeny of the *beddomei* clade (ND2 + 16S concatenated, 1610 base pairs) with photographs of the new species and *C.galaxia* (not to scale); numbers at nodes represent bootstrap support/ posterior probability > 70/0.99 (not shown close to terminal nodes). Inset, elevation map of the southern Western Ghats showing type and sampled localities for the *ornata* subclade.

The most diverse of the three subclades of the *beddomei* clade is the *ornata* subclade which includes nine valid species distributed from low elevations on the eastern slopes to high elevations (~ 200–1000+ m a.s.l.) in the Western Ghats south of Srivilliputhur (Fig. 1). Most species are low to mid elevation (~ 200–700 m a.s.l.) and are distributed on the eastern slopes as well as through some low passes onto the western slopes, and only *C.ornata* and the recently described *C.rashidi* are high elevation species found at elevations > 1,000 m a.s.l. (Sayyed et al. 2023a, b). Members of the *ornata* subclade are all strongly sexually dichromatic, diurnal, and scansorial, found on rocks, buildings and occasionally trees (Sayyed et al. 2019, 2023a, b; Pal et al. 2021; Khandekar et al. 2022). Seven of these species have been described since 2019, suggesting the diversity of this subclade is still incompletely known (Sayyed et al. 2019, 2023a, b; Pal et al. 2021; Khandekar et al. 2022).

As part of a project on the lizards of Tamil Nadu, we surveyed the southern Western Ghats from 2018–2022, specifically targeting known species of *Cnemaspis* as well as potential habitats that had not been previously sampled. We were able to collect most described species of the *ornata* subclade as well as multiple unnamed divergent lineages, two of which were subsequently described as *C.rashidi* and *C.sundara* (Sayyed et al. 2023a, b). In this paper, we provide molecular data from new localities for *C.galaxia*, *C.nairi*, *C.nigriventris*, *C.rashidi*, and *C.sundara* and describe two new species allied to *C.galaxia*. We also provide a brief note on the Code of Ethics and how it is rarely followed in the Indian context, and call for more collaborative research.

## ﻿Materials and methods

### ﻿Taxon sampling

Surveys were conducted in the early morning until a few hours after dark, specimens were observed on rocks, tree trunks, and collected by hand, followed by euthanasia using isoflurane after taking colour photos in life. Liver or tail tissues of at least two individuals of each new species/per locality were collected in molecular grade ethanol and subsequently stored at –20 °C for genetic analysis. Specimens were fixed in 8% formalin for ~ 12–24 h, washed and kept in tap water for ~ 24 h, and transferred to 70% ethanol for long-term storage. Collection permit was issued by the Tamil Nadu Forest Department (see acknowledgements), and collection protocols cleared by an inhouse ethics committee. Specimens are deposited in the Museum and Research Collection Facility at National Centre for Biological Sciences, Bengaluru (**NRC-AA**).

### ﻿Molecular data and analyses

We generated new sequences for 25 individuals representing five known species and three divergent lineages of the *ornata* subclade from ~ 18 localities (Fig. 1, Table 1). We targeted two mitochondrial genes that have been used in Indian *Cnemaspis* phylogenies, the protein coding ND2 and the large ribosomal subunit (16S). We extracted DNA from liver or tail-tips using the Qiagen DNeasy Blood and Tissue Extraction kit. We used the Macey et al. (1997) primers L4437 and H5934 to PCR amplify ND2 with L4437 and H5540 used for sequencing, and 16SA and 16SB (Palumbi et al. 1991) to amplify and sequence 16S; with PCR and sequencing outsourced to Barcode Biosciences, Bangalore. We combined the new sequences with published sequences for the *beddomei* clade using members of the *wynadensis* clade as outgroups (Table 1; Pal et al. 2021; Khandekar et al. 2022; Sayyed et al. 2023a, b). Sequences were aligned in MEGA 5.2 (Tamura et al. 2011) using CLUSTALW (Thompson et al. 1994) with default settings. The ND2 sequences were translated to amino acids to check for erroneous stop codons, which were absent, confirming we had sequenced the targeted mitochondrial protein coding gene. Pairwise uncorrected sequence divergence was calculated in MEGA 5.2 using the pairwise deletion option for each marker. We reconstructed phylogenetic relationships for the ND2 and 16S data separately (not shown as both mitochondrial markers were largely congruent) as well as in a concatenated analysis, using Maximum Likelihood (ML) in RaXML HPC 8.2.12 (Stamatakis 2014) and Bayesian Inference (BI) in MrBayes 3.2.7 (Ronquist and Huelsenbeck 2003). The best-fit models of sequence evolution and partitioning scheme were selected using the Bayesian Inference Criteria in PartitionFinder 2 (Lanfear et al. 2016) with the greedy algorithm (Lanfear et al. 2012) and RaxML (Stamatakis 2014). Three partitions were selected for each codon position of ND2 and one for 16S with the GTR+I+G model for codon position 1 and 16S and GTR+G for the other two codon positions. ML analyses employed 10 independent runs and 1000 non-parametric bootstraps (BS) to assess support. Partitioned BI analyses had parameters unlinked across partitions, four chains each (one cold and three hot) with two parallel runs with 1,000,000 generations sampled every 100 generations and convergence determined based on standard deviation of split frequencies (<< 0.01) and examination of ESS scores (> 200). The sumt function was used to build a consensus tree after removing the first 25% of trees as burn-in, with support assessed using posterior probability (PP) of each node.

**Table 1.** Sequences used in this study. Museum abbreviations are as follows: BNHS, Bombay Natural History Society, Mumbai; CESL, Centre for Ecological Sciences, Bangalore; NRCAA, National Centre for Biological Sciences, Bangalore; AK/ AK-R, Akshay Khandekar field series; AS, Amit Sayyed field series. All from India; KL = Kerala, MH = Maharashtra, TN = Tamil Nadu.

Species	Voucher	Locality	ND2	16S	Subclade
*Cnemaspisaaronbaueri*	AS 214	KL: Kollam District, Thenmala	OR714926	OR708521	*ornata*
*C.azhagu*	NRC-AA1171	TN: Tirunelveli District, Thirukurungudi range	–	PP382789	*ornata*
*C.azhagu*	NRC-AA1172	TN: Tirunelveli District, Thirukurungudi range	ON494554	PP382790	*ornata*
*C.galaxia*	AK-R 1323	TN: Virudhunagar District, Shenbaga Thopu	PP387688	PP382791	*ornata*
*C.galaxia*	AK-R 1324	TN: Virudhunagar District, Shenbaga Thopu	PP387689	PP382792	*ornata*
*C.galaxia*	AK-R 1422	TN: Virudhunagar District, Vyankateshpuram RF	PP387690	PP382793	*ornata*
*C.galaxia*	AK-R 1428	TN: Virudhunagar District, Shenbaga Thopu	–	PP382794	*ornata*
*C.galaxia*	AK-R 1470	TN: Virudhunagar District, Atti Kovil falls	PP387691	PP382795	*ornata*
*C.galaxia*	AK-R 1471	TN: Virudhunagar District, Atti Kovil falls	PP387692	PP382796	*ornata*
*C.galaxia*	AK-R 1473	TN: Virudhunagar District, Atti Kovil falls	PP387693	PP382797	*ornata*
*C.galaxia*	CESL 511	TN: Virudhunagar District, Shenbaga Thopu	MZ701818	MZ291589	*ornata*
*C.nairi*	AK-R 894	TN: Tenkasi District, Courtallam	PP387700	PP382804	*ornata*
*C.nairi*	AK-R 895	TN: Tenkasi District, Courtallam	PP387701	PP382805	*ornata*
*C.nairi*	CESL 712	KL: Kollam District, Shendurney	–	MZ291607	*ornata*
*C.nairi*	CESL 715	KL: Kollam District, Shendurney	–	MZ291608	*ornata*
*C.nigriventris*	AK-R 1265	TN: Tenkasi District, Courtallam	PP387702	PP382806	*ornata*
*C.nigriventris*	AK-R 1275	TN: Tenkasi District, Courtallam	PP387703	PP382807	*ornata*
*C.nigriventris*	CESL 264	KL: Kollam District, Achankovil RF	MZ291609	MZ701808	*ornata*
*C.nigriventris*	CESL 265	KL: Kollam District, Achankovil RF	MZ291610	–	*ornata*
*C.ornata*	CESL 276	TN: Tirunelveli District, Devarmalai Hills	MZ701809	MZ291613	*ornata*
*C.rashidi*	AK-R 1439	TN: Virudhunagar District, Srivilliputhur-Megamalai Tiger Reserve, higher elevations of Shenbaga Thopu	PP387704	PP382808	*ornata*
*C.rashidi*	AK-R 1440	TN: Virudhunagar District, Srivilliputhur-Megamalai Tiger Reserve, higher elevations of Shenbaga Thopu	PP387705	PP382809	*ornata*
*C.rashidi*	AK-R 1692	TN: Theni District, Srivilliputhur-Megamalai Tiger Reserve, Vellimalai	PP387706	PP382810	*ornata*
*C.rashidi*	AK-R 1693	TN: Theni District, Srivilliputhur-Megamalai Tiger Reserve, Vellimalai	PP387707	PP382811	*ornata*
*C.rashidi*	AS 226	TN: Virudhunagar District, Kottamalai estate	OR714921	–	*ornata*
*C.rashidi*	AS 228	TN: Virudhunagar District, Kottamalai estate	OR714922	–	*ornata*
*C.regalis*	CESL 487/ 488	TN: Tirunelveli District, Kalakad Mundanthurai Tiger Reserve	MZ701816/ MZ701817	MZ291615	*ornata*
*C.sathuragiriensis* sp. nov.	AK-R 1510	TN: Virudhunagar District, Sathuragiri Hills	PP387694	PP382798	*ornata*
*C.sathuragiriensis* sp. nov.	AK-R 1511	TN: Virudhunagar District, Sathuragiri Hills	PP387695	PP382799	*ornata*
*C.sundara*	AK-R 1254	TN: Tenkasi District, Mohan’s resort	PP387708	–	*ornata*
*C.sundara*	AK-R 1266	TN: Tenkasi District, Mohan’s resort	PP387709	–	*ornata*
*C.sundara*	BNHS 2916	TN: Tenkasi District, Mekkarai	OR714924	–	*ornata*
*C.sundara*	BNHS 2917	TN: Tenkasi District, Mekkarai	OR714925	–	*ornata*
*C.vangoghi* sp. nov.	AK-R 1351	TN: Virudhunagar District, Srivilliputhur-Megamalai Tiger reserve, Ayyanar Kovil,	PP387696	PP382800	*ornata*
*C.vangoghi* sp. nov.	AK-R 1352	TN: Virudhunagar District, Srivilliputhur-Megamalai Tiger reserve, Ayyanar Kovil,	PP387697	PP382801	*ornata*
*C.vangoghi* sp. nov.	AK-R 1373	TN: Virudhunagar District, Srivilliputhur-Megamalai Tiger reserve, Settur RF	PP387698	PP382802	*ornata*
*C.vangoghi* sp. nov.	AK-R 1374	TN: Virudhunagar District, Srivilliputhur-Megamalai Tiger reserve, Settur RF	PP387699	PP382803	*ornata*
*C.anamudiensis*	CESL 232	KL: Idukki District	MZ701805	MZ291574	*anamudiensis*
*C.nimbus*	CESL 252	KL: Idukki District, Mathikettan Shola NP	MZ701807	MZ291612	*anamudiensis*
*C.wallaceii*	CESL 377	TN: Coimbatore District, Anaimalai, Andiparai Shola	MZ701813	MZ291619	*anamudiensis*
*C.beddomei*	CESL 379	TN: Tirunelveli District, Kalakkad-Mundanthurai Tiger Reserve	MZ701814	MZ291581	*beddomei*
C.cf.maculicollis	CESL 709	KL: Kollam District, Shendurney WLS	MZ701825	MZ291582	*beddomei*
*C.rubraoculus*	CESL 114	KL: Idukki District, Periyar Tiger Reserve	ON494559	MZ291616	*beddomei*
*C.smaug*	CESL 251	KL: Idukki District, Mathikettan Shola NP	MZ701806	MZ291618	*beddomei*
*C.chengodumalaensis*	CESL 624	KL: Kozhikode District, Chengodumala	MZ701822	MZ291584	*wynadensis*
*C.kolhapurensis*	CESL 868	MH: Sindhudurg District, Amboli	MZ701829	MZ291599	*wynadensis*
*C.wynadensis*	CESL 630	KL: Wayanad District, Wayanad	MZ701823	MZ291620	*wynadensis*

### ﻿Morphological and meristic data

We restricted morphological comparisons to the *beddomei* clade (see Results). Morphological data were collected from 12 specimens of the two new species and from 42 specimens of the *beddomei* clade including type material of *C.azhagu*, *C.nimbus*, *C.smaug*, and *C.wallaceii*; type as well as topotypic and/ or additional materials for *C.galaxia*, *C.nigriventris*, *C.regalis*, and *C.rubraoculus*; and additional materials for *C.beddomei*, *C.nairi*, *C.rashidi*, and *C.sundara* (all listed in Appendix 1). Data for remaining four species— *C.aaronbaueri*, *C.anamudiensis*, *C.maculicollis*, and *C.ornata* (as well as *C.boiei* which is incertae sedis within *Cnemaspis*) were obtained from published literature (Manamendra-Arachchi et al. 2007; Cyriac et al. 2018; Sayyed et al. 2019; Pal et al. 2021). Meristic counts and measurements were taken under a ZEISS Stemi 305 stereo dissecting microscope by AK on the right side of the body where possible. Colour pattern was recorded from photographs taken in life. All measurements were taken with a Mitutoyo digital vernier calliper (to the nearest 0.1 mm). Mensural, meristic, and additional morphological character state evaluation is in accordance with Khandekar et al. (2019, 2024): snout vent length (SVL), tail length (TL), tail width (**TW**), forearm length (**FL**), crus length (**CL**), axilla to groin length (AGL), body height (**BH**), body width (**BW**), head length (**HL**), head width (HW), head depth (**HD**), eye diameter (**ED**), eye to ear distance (EE), eye to snout distance (**ES**), eye to nares distance (**EN**), internarial distance (**IN**), interorbital distance (**IO**), and ear length (**EL**); meristic data recorded for all specimens were number of supralabials (**SL**), infralabials (**IL**), supralabials at midorbital position (**SL M**), infralabials at midorbital position (IL M), paravertebral tubercles (**PVT**), dorsal tubercle rows (**DTR**), midventral scale rows across the belly (**MVSR**), ventral scales (**VS**), precloacal pores (**PP**), poreless scales between precloacal pores (SB PP), postcloacal tubercles (**PCT**), traverse distal subdigital lamellae on manus: digit 1 (**DLAMF1**), digit 4 (DLAMF4), on pes: digit 1 (**DLAMT1**), digit 4 (**DLAMT4**), and digit 5 (**DLAMT5**); traverse basal subdigital LAMellae on manus: digit 1 (BLAMF1), digit 4 (**BLAMF4**), on pes: digit 1 (**BLAMT1**), digit 4 (**BLAMT4**), and digit 5 (**BLAMT5**); total LAMellae (**TLAMF1**, **TLAMF4**, TLAMT1, **TLAMT4**, and TLAMT5). We follow Agarwal et al. (2020) for body size categories (SVL) for South Asian *Cnemaspis* (small bodied < 40 mm, medium-bodied 40–49 mm, large-bodied ≥ 50 mm).

## ﻿Results

### ﻿Phylogenetic relationships

**Figure 2.**
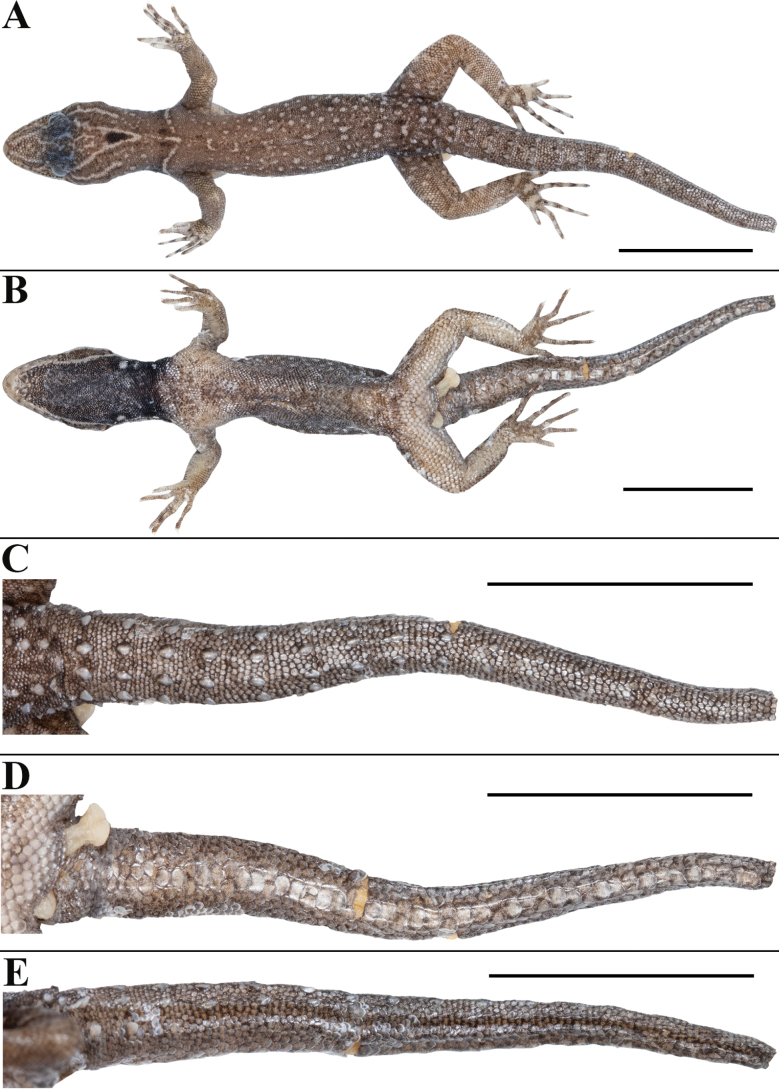
*Cnemaspisvangoghi* sp. nov. (holotype, NRC-AA-8342) **A** dorsal view of body **B** ventral view of body **C** dorsal view of tail **D** ventral view of tail **E** lateral view of tail. Photos by Akshay Khandekar. Scale bars: 10 mm.

We recovered the three subclades of the *beddomei* clade, *anamudiensis*, *beddomei*, and *ornata*, each of which received high support (Fig. 2; BS > 95, PP 1; Fig. 1). The two undescribed lineages fall within the *ornata* subclade and form a well-supported clade (BS 100, PP 1) together with *C.galaxia*. The two lineages have an uncorrected ND2 p-distance of 10.7% between each other (2.7% on 16S), 7.4–10.1% (3.1–3.4% 16S) from *C.galaxia*, and ≥ 15.6% (≥ 7.8% 16S) from all other members of the clade (Table 2). The lowest uncorrected ND2 p-distance between previously described species of the *ornata* subclade is 6.3% (2.2% 16S) between *C.nairi* and *C.nigriventris*. We describe the two genetically divergent lineages as new species below.

**Table 2.** Uncorrected % sequence divergence within the *C.ornata* subclade of the *beddomei* clade. Above diagonal, 16S; below diagonal, ND2; along diagonal in bold, maximum intraspecific ND2 divergence; – indicates no data.

		1	2	3	4	5	6	7	8	9	10	11
1	*C.sathuragiriensis* sp. nov.	–	2.7	–	7.3	3.4	12.8	12.0	12.6	14.2	8.1	–
2	*C.vangoghi* sp. nov.	10.7	**2.3**	–	8.4	3.1	12.9	12.4	12.7	14.2	9.0	–
3	*C.aaronbaueri*	19.4	17.6	–	–	–	–	–	–	–	–	–
4	*C.azhagu*	20.4	17.5	12.3	–	8.8	15.0	14.6	14.5	15.6	2.8	–
5	*C.galaxia*	10.1	7.4	19.6	18.2	**1.7**	12.7	12.1	12.5	14.7	9.0	–
6	*C.nairi*	31.1	30.3	28.7	29.8	30.9	**0.1**	2.2	7.2	10.0	15.3	–
7	*C.nigriventris*	31.2	29.8	28.8	29.9	30.1	6.3	**0.6**	7.7	9.7	15.0	–
8	*C.ornata*	29.4	28.4	28.1	28.8	29.0	21.6	19.0	–	4.8	15.2	–
9	*C.rashidi*	31.6	30.4	30.3	30.2	29.8	20.3	17.7	10.8	**3.0**	16.5	–
10	*C.regalis*	21.3	20.0	13.1	11.1	20.6	27.8	28.2	26.6	28.0	–	–
11	*C.sundara*	17.5	15.6	16.9	18.0	15.6	28.5	28.7	28.4	29.5	19.1	**3.0**

### ﻿Systematics

#### ﻿ Cnemaspis vangoghi sp. nov.

Taxon classificationAnimaliaSquamataGekkonidae

BAD23670-2D9A-53FE-8D01-F61DF964EB8E

https://zoobank.org/A27D1DEC-6E26-47A4-AEDC-69C139A6FE51

**Figure 3.**
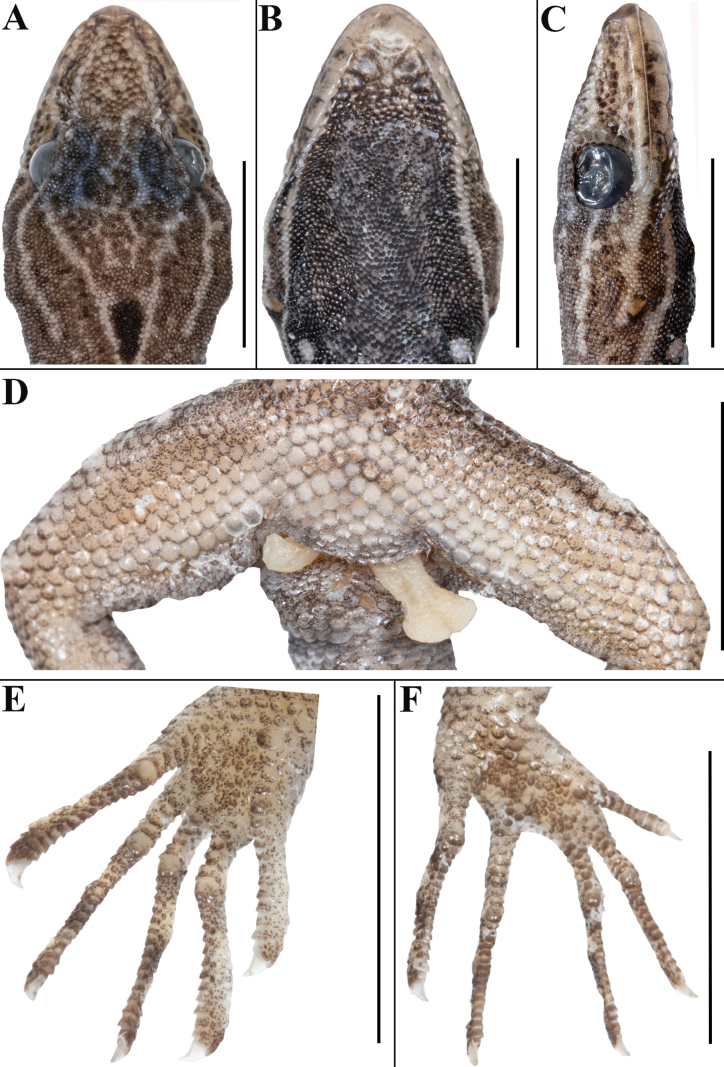
*Cnemaspisvangoghi* sp. nov. (holotype, NRC-AA-8342) **A** dorsal view of head **B** ventral view of head **C** lateral view of head on right **D** view of femoral region showing femoral pores **E** ventral view of left manus **F** ventral view of left pes. Photos by Akshay Khandekar. Scale bars: 5 mm.

**Figure 4.**
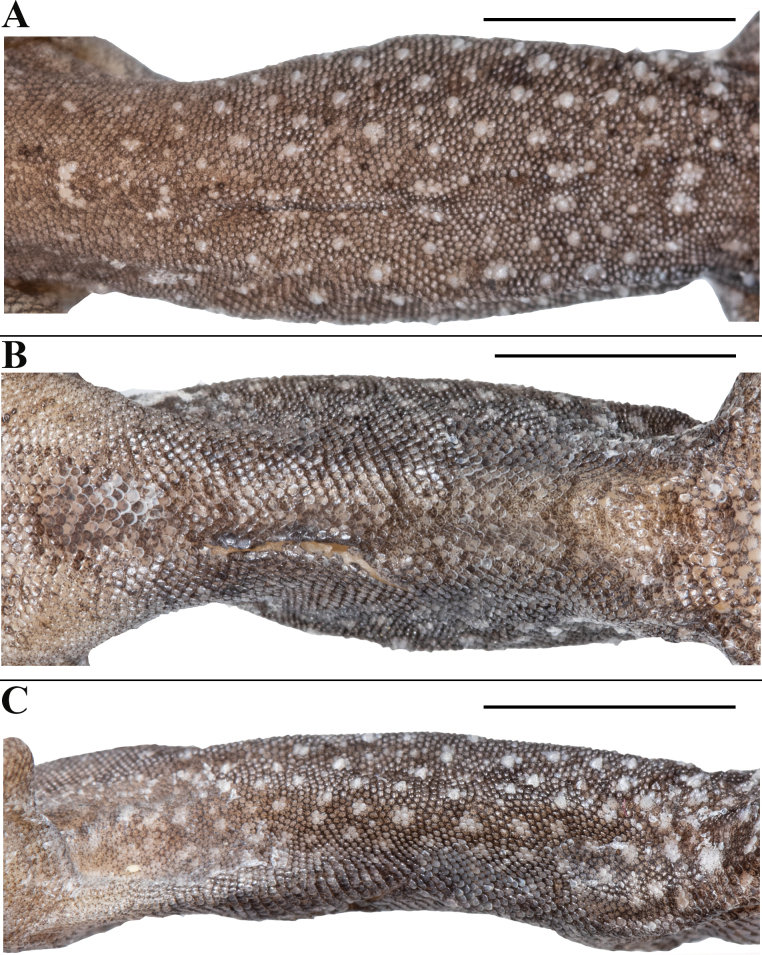
*Cnemaspisvangoghi* sp. nov. (holotype, NRC-AA-8342) **A** dorsal view of midbody **B** ventral view of midbody **C** lateral view of midbody. Photos by Akshay Khandekar. Scale bars: 5 mm.

**Figure 5.**
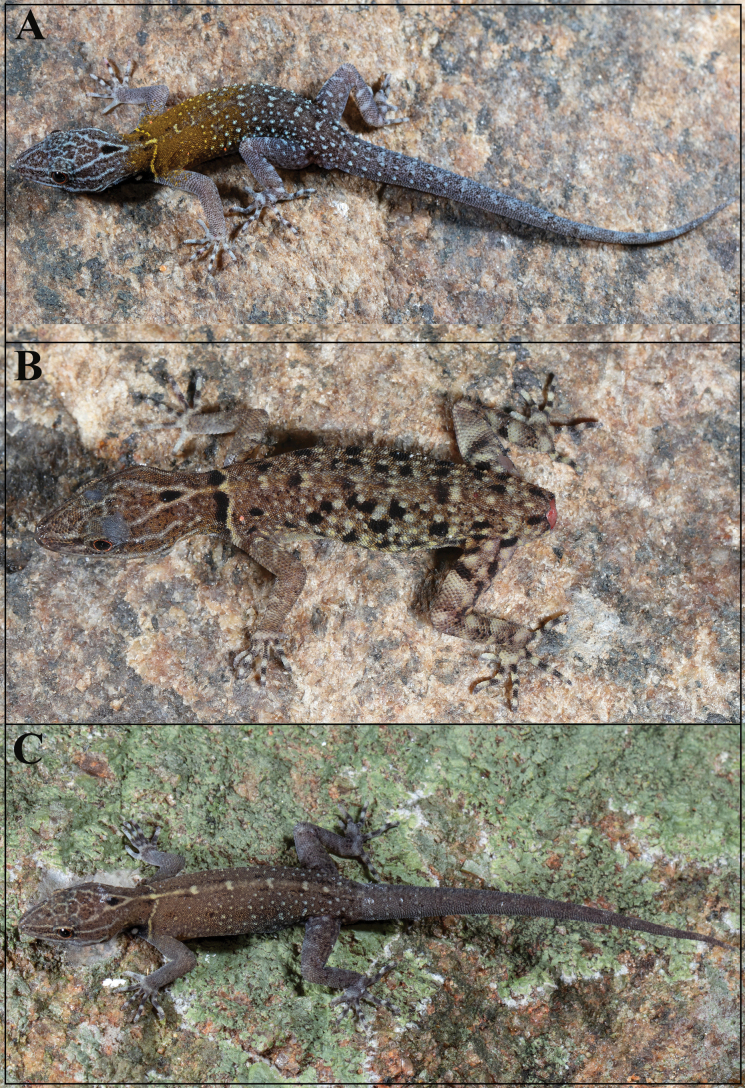
*Cnemaspisvangoghi* sp. nov., in life **A** adult male (holotype, NRC-AA-8342) **B** adult female (paratype, NRC-AA-8345), and **C** subadult male (paratype, NRC-AA-8348). Photos by Akshay Khandekar.

**Figure 6.**
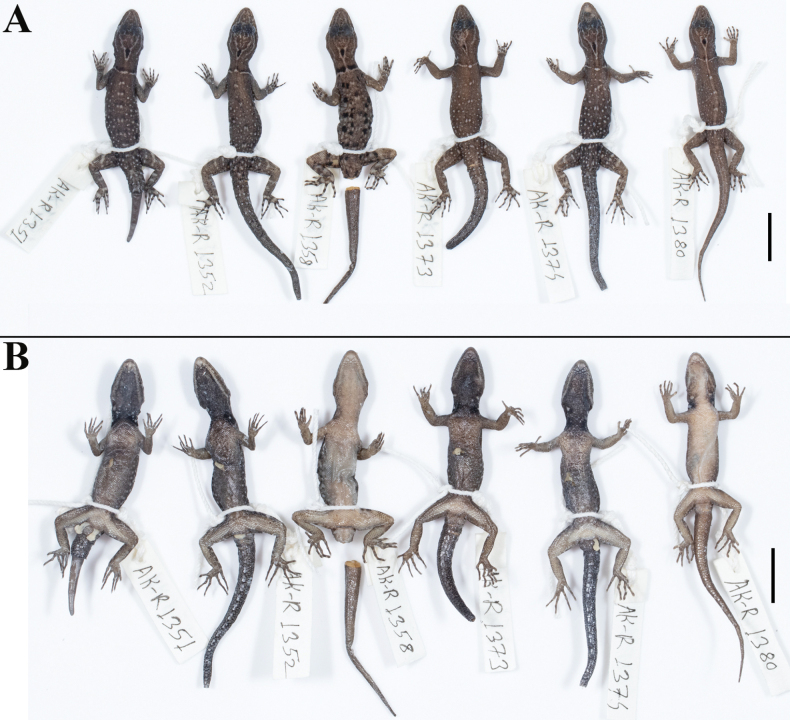
Paratype series of *Cnemaspisvangoghi* sp. nov., from left to right, NRC-AA-8343–8348 **A** dorsal view, and **B** ventral view. Photos by Akshay Khandekar. Scale bar: 10 mm.

Figs 2 , 3 , 4 , 5 , 6 , Tables 3 , 4 , 5

##### Type material examined.

***Holotype.***NRC-AA-8342 (AK-R 1356), adult male, from near Neer Katha Ayyanar Kovil (9.5108°N, 77.4529°E; ca 250 m a.s.l.), Srivilliputhur-Meghamalai Tiger Reserve, Virudhunagar district, Tamil Nadu state, India; collected by Akshay Khandekar, Ishan Agarwal, Swapnil Pawar and team on 16 April 2022. ***Paratypes.***NRC-AA-8343 (AK-R 1351), NRC-AA-8344 (AK-R 1352), adult males, same data as holotype; NRC-AA-8345 (AK-R 1358), adult female, from near Ayyanar Kovil waterfalls (9.5200°N, 77.4478°E; ca 400 m a.s.l.), same data as holotype; NRC-AA-8346 (AK-R 1373), NRC-AA-8347 (AK-R 1374), NRC-AA-8348 (AK-R 1380), adult males, from Settur Reserve Forest (9.4036°N, 77.3721°E; ca 350 m a.s.l.), same data as holotype except collected on 17 April 2022.

##### Diagnosis.

A small-sized *Cnemaspis*, snout to vent length ≤ 34 mm (*n* = 7). Dorsal pholidosis heterogeneous; smooth to weakly keeled granular scales intermixed with fairly regularly arranged rows of enlarged, weakly keeled, conical tubercles; 10 rows of dorsal tubercles at midbody, 7–14 tubercles in paravertebral rows; ventral scales subequal from chest to vent, smooth, subcircular and subimbricate with rounded end; 29–31 midventral scales across belly, 125–140 longitudinal ventral scales from mental to cloaca; subdigital scansors smooth, unnotched, some divided and others entire, a distinct enlarged metacarpal scale below digit I; 11–14 lamellae under digit I of manus and 11–13 under digit I of pes, 19–22 lamellae under digit IV of manus and 18–25 lamellae under digit IV of pes; males with continuous series of six or seven precloacal pores (*n* = 6); scales on non-regenerated tail dorsum heterogeneous; small, smooth, subcircular, flattened, subimbricate scales intermixed on anterior one third portion with enlarged, weakly keeled, and weakly conical tubercles forming seven whorls; six tubercles on first three whorl, four tubercles on fourth to seventh whorls, only a pair of paravertebral tubercles each on eighth to 11^th^ whorls; rest of the tail lacking enlarged tubercles; median row of subcaudals smooth, roughly rectangular, distinctly enlarged, with condition of two enlarged scales alternating with a divided scale. Males with ochre anterior 1/2 of body, single central black dorsal ocellus on neck, a white ocellus on ventrolateral side of neck and one on throat posterior to jaw, venter off-white with dark throat, tail unbanded, females and juveniles brown, juveniles with indistinct mid-dorsal streak.

##### Comparisons with members of *beddomei* clade.

*Cnemaspisvangoghi* sp. nov. can be easily distinguished from all 16 members of the *beddomei* clade as well as from *C.boiei* by a combination of the following differing or non-overlapping characters: A small-sized *Cnemaspis*, snout to vent length ≤ 34 mm (vs medium-sized *Cnemaspis*, snout to vent length 40–49 mm in *C.nairi*, *C.nimbus*, *C.ornata*, *C.rashidi*, *C.rubraoculus*, and *C.wallaceii*; large-sized *Cnemaspis*, snout to vent length > 50 mm in *C.anamudiensis*, *C.beddomei*, *C.maculicollis*, and *C.smaug*; snout to vent length ≤ 38 mm in *C.azhagu*, *C.boiei*, and *C.nigriventris*); ten rows of dorsal tubercles at midbody (vs only a few enlarged scattered tubercles at midbody dorsum in *C.anamudiensis*, two or three rows of dorsal tubercles at midbody in *C.azhagu*, eight in *C.galaxia*, 16–18 in *C.nairi*, 13 or 14 in *C.nigriventris*, 12–14 in *C.nimbus* and *C.ornata*, 7–9 in *C.regalis*, 19–22 in *C.smaug*, six in *C.sundara*, 14 or 15 in *C.wallaceii*); 125–140 longitudinal ventral scales from mental to cloaca (vs 151–171 longitudinal ventral scales from mental to cloaca in *C.azhagu*, 154–161 in *C.beddomei*, 153–159 in *C.galaxia*, 143–147 in *C.nairi*, 154–159 in *C.nigriventris*, 157–165 in *C.ornata*, 170–172 in *C.rashidi*, 148–154 in *C.regalis*, 142–150 in *C.smaug*, 156–160 in *C.sundara*, 154–156 in *C.wallaceii*); 7–14 tubercles in paravertebral rows (vs paravertebral tubercles either absent or irregular in *C.anamudiensis*, *C.azhagu*, and *C.sundara*, 18 or 19 tubercles in paravertebral rows in *C.aaronbaueri* and *C.beddomei*, 16 or 17 in *C.nimbus*, 21–23 in *C.ornata*, 27–30 in *C.smaug*, 18–20 in *C.wallaceii*); 29–31 midventral scales across belly (vs 34–44 midventral scales across belly in *C.azhagu*, 32 or 33 in *C.nairi*, 38–40 in *C.nigriventris*, 26 or 27 in *C.nimbus*, 40–44 in *C.regalis*, 33–37 in *C.rubraoculus*, 35 or 36 in *C.sundara*); a distinct white ocellus on ventrolateral sides of neck present in males (vs white ocellus on ventrolateral sides of neck absent in *C.aaronbaueri*, *C.anamudiensis*, *C.azhagu*, *C.beddomei*, *C.maculicollis*, *C.nimbus*, *C.nimbus*, *C.regalis*, *C.rubraoculus*, *C.smaug*, *C.wallaceii*); tail unbanded (tail distinctly banded in *C.nairi*, *C.nigriventris*, *C.ornata*, *C.rashidi*, *C.smaug*, *C.sundara*). *Cnemaspisvangoghi* sp. nov. is diagnosed against the other new species as part of its description below.

##### Description of the holotype.

Adult male in good state of preservation except tail marginally bent towards left and tip is missing, hemipenis partially everted on right and fully on left side, and a 3.1 mm long incision in sternal region for tissue collection (Fig. 2A, B); SVL 32.1 mm, head short (HL/SVL 0.25), wide (HW/HL 0.68), not strongly depressed (HD/HL 0.40), distinct from neck. Loreal region marginally inflated, canthus rostralis indistinct. Snout 1/2 head length (ES/HL 0.48), 2.5× eye diameter (ES/ED 2.5); scales on snout and canthus rostralis subcircular to elongate, subequal, smooth, weakly conical, much larger than those on forehead and interorbital region; scales on forehead similar to those on snout and canthus rostralis except almost 2× smaller and elongate; scales on interorbital region, occipital, and temporal region even smaller, granular (Fig. 3A). Eye small (ED/HL 0.19); with round pupil; supraciliaries short, larger anteriorly; eight interorbital scale rows across narrowest point of frontal bone; 27 scale rows between left and right supraciliaries at mid-orbit level (Fig. 3A). Ear-opening deep, oval, small (EL/ HL 0.06); eye to ear distance much greater than diameter of eye (EE/ED 1.60) (Fig. 3C). Rostral slightly > 2× as wide (1.5 mm) as high (0.7 mm), incompletely divided dorsally by a strongly developed rostral groove for > 1/2 of its height; a single enlarged, roughly rectangular supranasal on each side, almost 3× larger than upper postnasal, and strongly in contact with each other on snout; a pair of enlarged scales on snout behind internasals, separated from each other by two much smaller, granular scales; rostral in contact with supralabial I, nostril, and supranasal on either side; nostrils oval, surrounded by four postnasals, supranasal, rostral and supralabial I on either side; four roughly circular postnasals on either side, the one touching supranasal largest, gradually decreasing in side posteriorly; two single row of scales separate orbit from supralabials (Fig. 3C). Mental enlarged, subtriangular, marginally wider (2.0 mm) than high (1.6 mm); two pairs of postmentals, inner pair roughly rectangular, shorter (0.9 mm) than mental, separated from each other below mental by a single enlarged median chin shield; inner pair bordered by mental, infralabial I, outer postmental, median chin shield and a single enlarged chin shields on either side; outer postmentals roughly rectangular, slightly smaller (0.6 mm) than inner pair, bordered by inner postmentals, infralabial I and II, and four enlarged chin shields on either side; three enlarged gular scales between left and right outer postmentals; all chin scales bordering postmentals more or less flattened, subcircular, smooth, and smaller than outermost postmentals; scales on rest of throat, much smaller, smooth, subcircular, and subimbricate (Fig. 3B). Infralabials bordered below by a row or two of slightly enlarged, much elongated scales, decreasing in size posteriorly. Nine supralabials up to angle of jaw and five at midorbital position on each side; supralabial I largest, gradually decreasing in size posteriorly; eight infralabials on left and seven on right side up to angle of jaw, four at midorbital position on left and five on right side; infralabial I largest, gradually decreasing in size posteriorly (Fig. 3C).

Body relatively slender (BW/AGL 0.37), trunk < 1/2 of SVL (AGL/SVL 0.42) without spine-like tubercles on flank (Fig. 4A–C). Dorsal pholidosis heterogeneous; smooth to weakly keeled granular scales intermixed with a fairly regularly arranged rows of enlarged, weakly keeled, conical tubercles; granular scales gradually increasing in size towards each flank, largest on mid-flank; granular scales on occiput and nape slightly smaller than paravertebral granules; enlarged tubercles in approximately 10 longitudinal rows at midbody; 12 (left) and 14 (right) tubercles in paravertebral rows (Fig. 4A, C). Ventral scales much larger than granular scales on dorsum, subequal from chest to vent, smooth, subcircular and subimbricate with rounded end; scales on precloacal region and four or five rows on femur distinctly enlarged; midventral scale rows across belly 31; 138 ventral scales from mental to anterior border of cloaca (Fig. 4B). A continuous series of six precloacal pores, femoral pores absent (Fig. 3D).

Scales on palm and soles granular, smooth, rounded, and flattened, a distinct enlarged metacarpal scale on palm below digit I; scales on dorsal aspects of limbs heterogeneous in shape and size; scales on upper arm and thigh much larger than granular scales on body dorsum, elongate, subimbricate with pointed ends; scales on lower arm and shank granular, similar in size to granular scales on body dorsum, smooth, rounded, gradually becoming larger, flattened and subimbricate anterolaterally and posteriorly, largest on anterolateral aspect of the hands and feet; scales on ventral aspect of upper arm smooth, granular, much smaller than granular scales on body dorsum, scales on ventral aspect of lower arm with much larger scales than those on upper arm, smooth, subcircular and flattened scales; ventral aspect of thigh and shank with enlarged, smooth, flattened, subimbricate scales, much larger than midventrals (Fig. 2A, B). Forelimbs and hindlimbs slightly long, slender (LAL/ SVL 0.14); (CL/SVL 0.19); digits long, with a strong, recurved claw, distinctly inflected, distal portions laterally compressed conspicuously. Digits with both paired and unpaired lamellae, separated into a basal and narrower distal series by single enlarged lamella at inflection; one or two most basal paired on basal series and 1–4 paired lamellae above the inflection; basal lamellae series: (1-5-5-6-5 right manus, 2-7-7-7-4 right pes), (2-6-5-6-5 left manus, Fig. 3E; 2–7–7–8–3 left pes, Fig. 3F); distal lamellae series: (11-12-16-15-12 right manus, 10-12-16-16-16 right pes), (11-12-15-15-12 left manus, Fig. 3E; 10–12–16–16–16 left pes, Fig. 3F). Relative length of digits (measurements in mm in parentheses): IV (3.2) > III (3.1) > II (2.9) > V (2.7) > I (2.1) (left manus); IV (4.1) > V (3.9) = III (3.6) > II (2.9) > I (1.9) (left pes).

Tail original, subcylindrical, slender, not entire, tail tip is detached and missing, TL = 27.2 mm (Fig. 2C–E). Dorsal pholidosis on tail heterogeneous; small, smooth, subcircular, flattened, subimbricate scales intermixed on anterior one third portion with enlarged, weakly keeled, and weakly conical tubercles forming seven whorls; six tubercles on first three whorl, four tubercles on fourth to seventh whorls, only a pair of paravertebral tubercles on 8^th^ to 11^th^ whorls; rest of the tail lacking enlarged tubercles (Fig. 2C, E). Scales on tail venter much larger than those on dorsal aspect, smooth, roughly subcircular, flattened, subimbricate; median series smooth, roughly rectangular, distinctly enlarged than rest, with condition of two enlarged scales alternating with a divided scale (Fig. 2D). Scales on tail base much smaller, smooth, imbricate; a single enlarged, smooth and weakly conical postcloacal tubercle on each side (Fig. 2D, E).

##### Colouration in life

**(Fig. 5A).** Dorsal ground colour of body, limbs and tail light grey; neck to mid-body ochre, fading slightly at mid-body. Light blue-grey preorbital streak runs from nostril to orbit; three light postorbital streaks, uppermost on either side meeting in parietal region forming an inverted chevron enclosing a single large elongate black ocellus on occiput, middle terminating on neck and lowermost continuing until ear opening. Head finely reticulated with pale blue-grey, a white ocellus on a black patch of scales on each side of ventrolateral aspect of neck just anterior to forelimb insertions; a fine yellow collar at anterior edge of forelimb insertions, just divided by indistinct continuation of chevron on neck, two small black spots anterior to the division. No distinct dorsal spots or bands, tubercles and a few adjacent scales at mid-body and posterior 1/2 of body pale blue-grey; similar spots on femur and bands on tibia; forelimbs with some ochre near insertions, otherwise whitish-grey with dark outlines of scales; digits with white and dark markings. Original tail without bands, blue-grey with dark outlines of scales. Ventral ground colouration grey-white; throat strongly marked with black up to forelimb insertions except for a fine pale border just below infralabials; a white spot on either side of the throat posterior to jaw; belly with dark markings and blue-grey scales toward the lateral margins; underside of limbs and tail with few dark markings; precloacal, femoral and tibial regions with almost no dark markings. Pupil black, iris reddish with a pale orange ring lining pupil.

##### Variation and additional information from type series

**(Figs 5B, C, 6).** Mensural, meristic and additional character state data for the type series is given in Tables 3–5, respectively. There are four adult males, a single subadult male, and a single adult female ranging in size from 28.6–33.6 mm (Fig. 6A, B). All paratypes resemble the holotype except as follows: three postnasals on either side in NRC-AA-8344, NRC-AA-8346, and NRC-AA-8348. Inner postmentals bordered by mental, infralabial I, outer postmental, enlarged median chin shield in all paratypes, additionally, bordered by two small chin scales on either side in NRC-AA-8343, single chin scale on left and two on right side in NRC-AA-8344. Outer postmentals bordered by inner pair, infralabial I and II in all paratypes, additionally, bordered by five chin scales on left and four on right side in NRC-AA-8344, NRC-AA-8345, NRC-AA-8347; four on left and five on right side in NRC-AA-8348; outer postmental separated from each other by five chin scales including median chin shield in NRC-AA-8343, four chin scales in NRC-AA-8344. NRC-AA-8348 with original and complete tail, slightly longer than body (TL/SVL 1.23); three paratypes, NRC-AA-8344, NRC-AA-8346, and NRC-AA-8347, with original partially broken tails; NRC-AA-8343 with small and partially regenerated tail, and NRC-AA-8345 with complete regenerated tail, detached from the body (Fig. 6A, B). NRC-AA-8347 with damaged skink on the snout; NRC-AA-8343 with fully everted hemipenis on either side, NRC-AA-8347 with fully everted hemipenis only on left side.

**Table 3.** Mensural (mm) data for the new species. Abbreviations are listed in Materials and methods, * = tail incomplete.

Museum number	*Cnemaspisvangoghi* sp. nov.							*Cnemaspissathuragiriensis* sp. nov.				
	Holotype	Paratypes						Holotype	Paratypes			
	NRC-AA-8342	NRC-AA-8343	NRC-AA-8344	NRC-AA-8345	NRC-AA-8346	NRC-AA-8347	NRC-AA-8348	NRC-AA-8349	NRC-AA-8350	NRC-AA-8351	NRC-AA-8352	NRC-AA-8353
Sex	Male	Male	Male	Female	Male	Male	Subadult male	Male	Male	Male	Male	Subadult female
SVL	32.1	33.6	33.6	32.9	31.3	32.8	28.6	32.8	32.9	31.2	33.0	26.7
TL	27.2*	16.9*	29.8*	33.7*	22.2*	26.9*	35.4	37.6	38.6	32.1*	3.1*	1.9*
TW	3.2	3.1	3.2	3.4	3.3	3.0	2.7	3.0	2.7	3.6	3.2	2.3
FL	4.7	4.6	5.0	5.2	4.9	4.7	4.6	4.8	4.9	4.9	5.1	4.0
CL	6.2	5.9	6.3	6.2	6.3	6.2	5.3	6.5	6.2	6.0	6.7	4.9
AGL	13.7	13.0	12.8	13.2	12.6	13.6	11.5	12.9	14.2	12.4	12.7	11.1
BH	3.5	3.7	3.4	4.2	3.6	3.4	3.7	3.6	3.5	3.3	4.2	3.9
BW	5.2	7.0	5.9	6.3	6.2	5.7	5.6	6.5	5.6	6.1	6.9	5.1
HL	8.2	8.5	8.8	9.1	8.4	8.8	7.0	9.0	8.1	8.5	8.9	6.6
HW	5.6	6.1	5.9	5.9	5.8	5.7	5.4	6.7	5.9	6.1	5.9	4.7
HD	3.3	3.5	3.7	4.6	3.78	3.7	3.3	4.7	3.4	3.7	3.8	2.7
ED	1.6	1.6	1.9	1.8	1.6	1.5	1.4	1.8	1.7	1.8	1.9	1.5
EE	2.6	2.9	2.7	2.8	2.8	2.9	2.3	2.9	2.6	2.7	2.7	2.3
ES	4.0	3.9	4.3	4.3	3.9	4.2	3.3	4.0	4.0	4.0	4.3	3.1
EN	3.1	3.1	3.4	3.2	3.1	3.3	2.6	3.2	3.0	3.2	3.5	2.5
IN	1.1	1.1	1.1	1.2	1.1	1.0	0.8	1.1	1.0	1.1	1.2	1.0
IO	2.1	2.3	2.5	2.6	2.5	2.1	2.1	2.4	2.4	1.8	2.3	1.9
EL	0.5	0.6	0.5	0.6	0.8	0.5	0.5	0.5	0.7	0.6	0.7	0.4

**Table 4.** Meristic data for the new species. Abbreviations are listed in Materials and methods, * = lamellae damaged, L&R = left & right, A = absent.

Museum number	*Cnemaspisvangoghi* sp. nov.							*Cnemaspissathuragiriensis* sp. nov.				
	Holotype	Paratypes						Holotype	Paratypes			
	NRC-AA-8342	NRC-AA-8343	NRC-AA-8344	NRC-AA-8345	NRC-AA-8346	NRC-AA-8347	NRC-AA-8348	NRC-AA-8349	NRC-AA-8350	NRC-AA-8351	NRC-AA-8352	NRC-AA-8353
SL L&R	9&9	9&8	9&9	8&8	7&7	9&10	8&7	8&8	8&8	8&8	8&8	8&8
IL L&R	8&7	6&7	7&7	8&7	6&7	7&7	8&8	7&7	7&7	7&7	7&7	7&7
SL M L&R	5&5	5&5	6&5	5&6	5&5	5&6	5&5	5&5	5&5	5&5	5&5	5&5
IL M L&R	4&5	4&4	5&5	4&5	5&4	4&5	4&4	5&5	4&4	4&4	4&4	5&4
PVT L&R	12&14	7&7	7&8	7&9	12&12	11&13	7&7	A	A	irr	A	irr
DTR	10	10	10	10	10	10	10	6	7	8	6	7
MVSR	31	31	30	30	29	29	31	30	28	30	28	29
VS	138	125	137	126	134	140	134	132	131	137	130	131
DLAMF1 L&R	11&11	10&10	11&11	9&10	10&11	12&12	12&12	10&10	10&10	10&10	10&10	11&12
BLAMF1 L&R	2&1	2&2	2&2	2&2	2&2	2&2	2&1	1&1	1&1	3&3	2&2	1&1
DLAMF4 L&R	15&15	13&13	14&15	13&14	14&14	16&10*	15&15	14&14	15&14	15&15	13&13	15&15
BLAMF4 L&R	6&6	6&6	6&6	6&6	6&6	6&6	7&6	6&6	3&4	6&6	6&6	6&5
DLAMT1 L&R	10&10	9&9	10&10	9&9	10&10	11&11	11&10	10&10	10&10	9&9	9&9	10&10
BLAMT1 L&R	2&2	2&2	2&2	2&2	2&2	2&2	2&2	1&1	2&2	2&2	2&2	2&2
DLAMT4 L&R	16&16	15&15	15&15	14&15	14&14	17&16	16&17	15&16	16&16	16&16	14&14	16&16
BLAMT4 L&R	8&7	7&6	6&6	4&6	6&7	8&8	7&7	8&8	7&7	8&8	9&9	7&7
DLAMT5 L&R	16&16	15&14	15&15	15&14	15&15	17&16	16&17	15&16	15&16	15&16	13&14	16&16
BLAMT5 L&R	3&4	2&2	2&2	2&2	3&3	2&2	2&2	2&2	2&2	2&2	2&3	2&2
TLAMF1 L&R	13&12	12&12	13&13	11&12	12&13	14&14	14&13	11&11	11&11	13&13	12&12	12&13
TLAMF4 L&R	21&21	19&19	20&21	19&20	20&20	22&16*	22&21	20&20	18&18	21&21	19&19	21&20
TLAMT1 L&R	12&12	11&11	12&12	11&11	12&12	13&13	13&12	11&11	12&12	11&11	11&11	12&12
TLAMT4 L&R	24&23	22&21	21&21	18&21	20&21	25&24	23&24	23&24	23&23	24&24	23&23	23&23
TLAMT5 L&R	19&20	17&16	17&17	17&16	18&18	19&18	18&19	17&18	17&18	17&18	15&17	18&18
PP	6	7	7	A	7	7	7	7	8	7	8	A
SB PP	A	A	A	A	A	A	A	A	A	A	A	A
PCT L&R	1&1	1&1	1&1	1&1	1&1	1&1	1&1	1&1	1&1	1&1	1&1	1&1

**Table 5.** Additional morphological characters of the new species. A = absent, / = data unavailable.

Museum number	*Cnemaspisvangoghi* sp. nov.							*Cnemaspissathuragiriensis* sp. nov.				
	Holotype	Paratypes						Holotype	Paratypes			
	NRC-AA-8342	NRC-AA-8343	NRC-AA-8344	NRC-AA-8345	NRC-AA-8346	NRC-AA-8347	NRC-AA-8348	NRC-AA-8349	NRC-AA-8350	NRC-AA-8351	NRC-AA-8352	NRC-AA-8353
Anterior extra-brillar fringe scales enlarged (0) or not enlarged (1)	0	0	0	0	0	0	0	0	0	0	0	0
Occipital ocellus present (0) or absent (1)	0	0	0	0	0	0	0	0	0	0	0	0
Dorsal pholidosis homogeneous (0) or heterogeneous (1)	1	1	1	1	1	1	1	1	1	1	1	1
Dorsal tubercles weakly keeled (0) or smooth (1)	0	0	0	0	0	0	0	0	0	0	0	0
Tubercles linearly arranged (0) or more random (1)	0	0	0	0	0	0	0	1	1	1	1	1
Spine-like tubercles on flank present (0) or absent (1)	1	1	1	1	1	1	1	1	1	1	1	1
Gular scales keeled (0) or smooth (1)	1	1	1	1	1	1	1	1	1	1	1	1
Pectoral scales keeled (0) or smooth (1)	1	1	1	1	1	1	1	1	1	1	1	1
Ventral scales keeled (0) or smooth (1)	1	1	1	1	1	1	1	1	1	1	1	1
Precloacal pores continuous (0) or separated (1)	0	0	0	A	0	0	0	0	0	0	0	A
Precloacal pores elongate (0) or round (1)	0	0	0	A	0	0	0	0	0	0	0	A
Enlarged femoral scales present (0) or absent (1)	0	0	0	0	0	0	0	0	0	0	0	0
Subtibial scales keeled (0) or smooth (1)	1	1	1	1	1	1	1	1	1	1	1	1
Lateral caudal furrows present (0) or absent (1)	1	/	1	/	1	1	1	1	1	1	/	/
Caudal tubercles encircle tail (0) or not (1)	1	/	1	/	1	1	1	1	1	1	/	/
Subcaudals keeled (0) or smooth (1)	1	/	1	/	1	1	1	1	1	1	/	/
Median subcaudal scale row not enlarged (0) or enlarged (1)	1	/	1	/	1	1	1	1	1	1	/	/

The new species is strongly sexually dimorphic and also shows ontogenetic colour variation (Fig. 5A–C): females brown with numerous black and pale blotches, collar pale brown, flanked anteriorly by thick black, divided by an extension of the neck chevron; distinct black ocellus on occiput; white ocelli on side of neck absent; forelimbs brown, hindlimbs with scattered dark and pale markings, digits banded. Regenerated tail grey, without bands. Ventral ground colouration of gular, body and tail grey-white; underside of limbs with few dark markings. Subadult male brown with an indistinct, cream mid-dorsal streak formed by the extension of the neck chevron, five or six spots in the streak; black ocellus on occiput and white ocelli on side of neck distinct; forelimbs brown, hindlimbs with scattered dark and pale markings, digits banded. Original tail without bands, grey with dark outlines of scales, regenerated portion brown. Ventral ground colouration of gular, body and tail grey-white; a white spot on either side of the throat posterior to jaw; belly without dark markings; underside of limbs and tail with few dark markings.

##### Etymology.

The specific epithet is a patronym for Dutch painter Vincent Van Gogh (1853–1890). The colouration of the new species is reminiscent of one of Van Gogh’s most iconic paintings, The Starry Night. Suggested common name is Van Gogh’s starry dwarf gecko.

##### Distribution and natural history.

**Figure 7.**
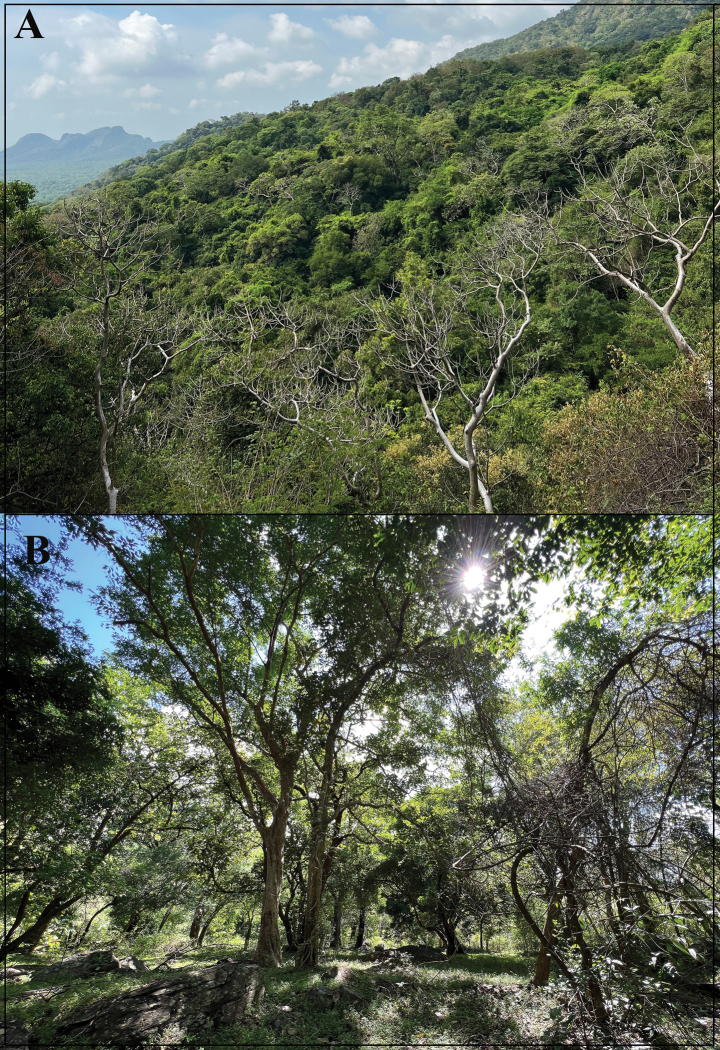
Habitat of *Cnemaspisvangoghi* sp. nov. **A** general view at Settur Reserve Forest (paratype locality), and **B** microhabitat at type locality. Photos by Akshay Khandekar.

*Cnemaspisvangoghi* sp. nov. is known only from two closely spaced localities (Ayyanar Kovil and Settur Reserve Forest, both in Meghamalai-Srivilliputhur Tiger Reserve, Tamil Nadu) within < 15 km straight line distance (Fig. 1). The new species was recorded in seasonally dry tropical forest with a mix of evergreen and deciduous species between elevations of 250–400 m a.s.l. on eastern slopes of the Western Ghats (Fig. 7A). Individuals of the new species were observed active during the daytime (0830–1400 hrs) on rocks and tree trunks < 2 m high from the base (Fig. 7B). A large number of individuals (*n* ≥ 25/hr) were observed at both the locations indicating high abundance. At Ayyanar Kovil, a few individuals were observed inactive, resting on rocks during evening and night time (1800–2030 hrs). We also observed Giant wood spider (*Nephila* sp.) feeding on an adult female individual of the new species. Sympatric lizards at the type locality include Cnemaspiscf.gracilis, Hemidactyluscf.frenatus, H.cf.leschenaultii, *H.vanam* Chaitanya, Lajmi & Giri, 2018, *Dravidosepssrivilliputhurensis* Agarwal, Thackeray & Khandekar, 2024, *Eutropiscarinata* (Schneider, 1801), *E.macularia* (Blyth, 1853), and Psammophiluscf.blanfordanus.

#### ﻿ Cnemaspis sathuragiriensis sp. nov.

Taxon classificationAnimaliaSquamataGekkonidae

3A6B75DA-8088-5605-96FD-635AD7C17276

https://zoobank.org/5C640413-8B2F-417F-AD1C-A867AFE5A7B5

**Figure 8.**
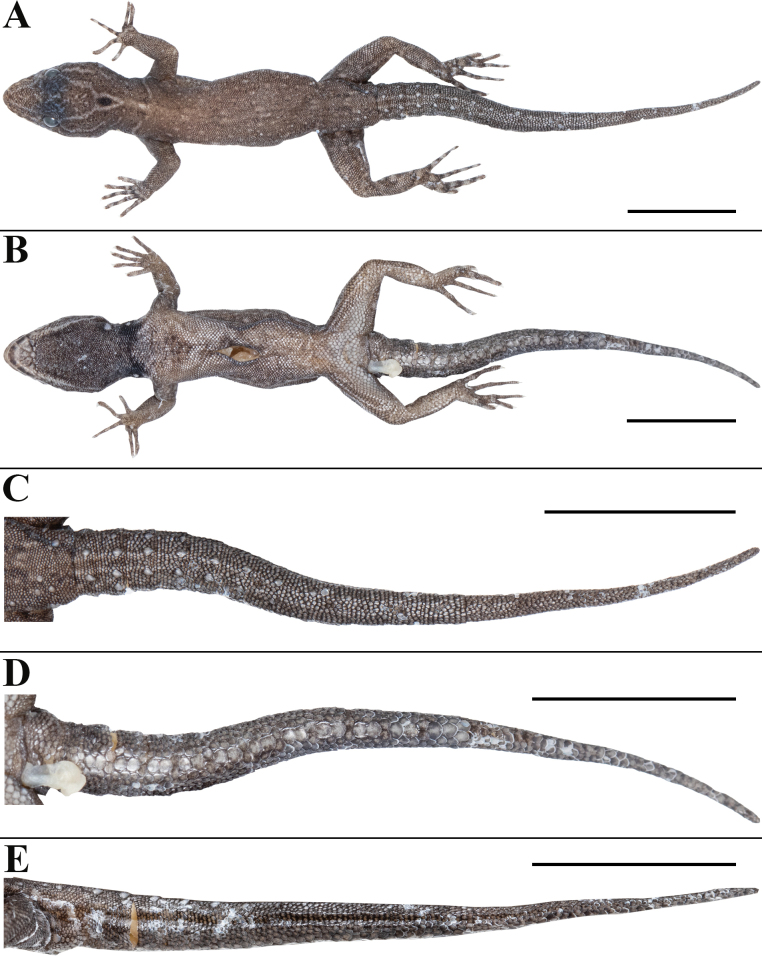
*Cnemaspissathuragiriensis* sp. nov. (holotype, NRC-AA-8349) **A** dorsal view of body **B** ventral view of body **C** dorsal view of tail **D** ventral view of tail **E** lateral view of tail. Photos by Akshay Khandekar. Scale bars: 10 mm.

**Figure 9.**
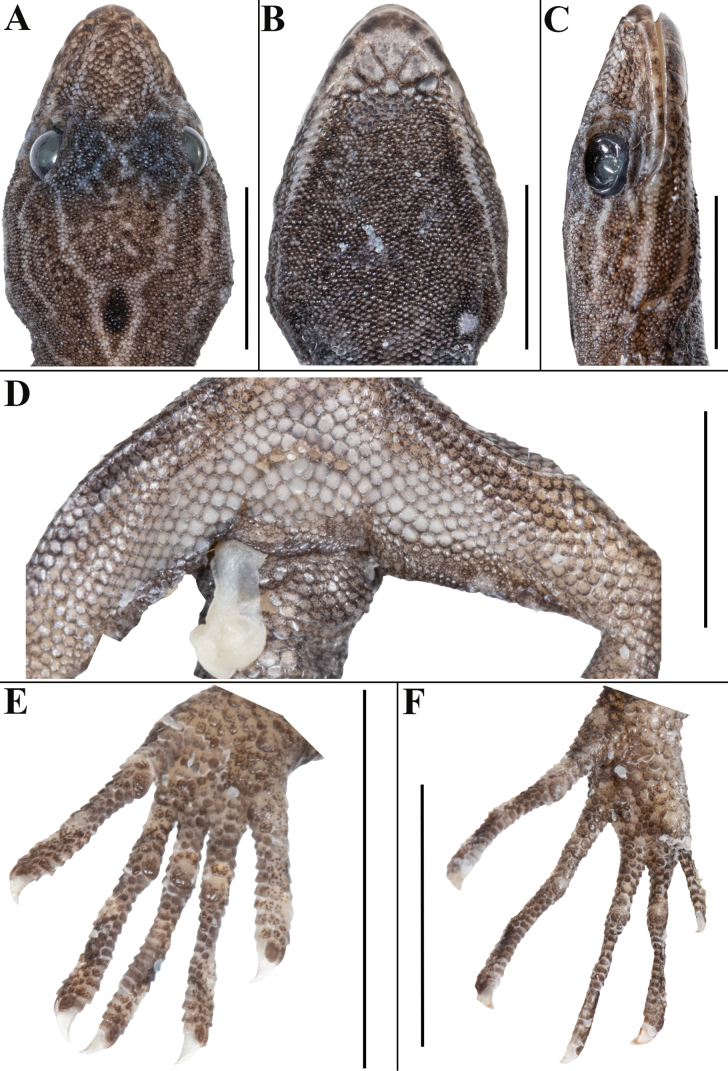
*Cnemaspissathuragiriensis* sp. nov. (holotype, NRC-AA-8349) **A** dorsal view of head **B** ventral view of head **C** lateral view of head on right **D** view of femoral region showing femoral pores **E** ventral view of left manus **F** ventral view of left pes. Photos by Akshay Khandekar. Scale bars: 5 mm.

**Figure 10.**
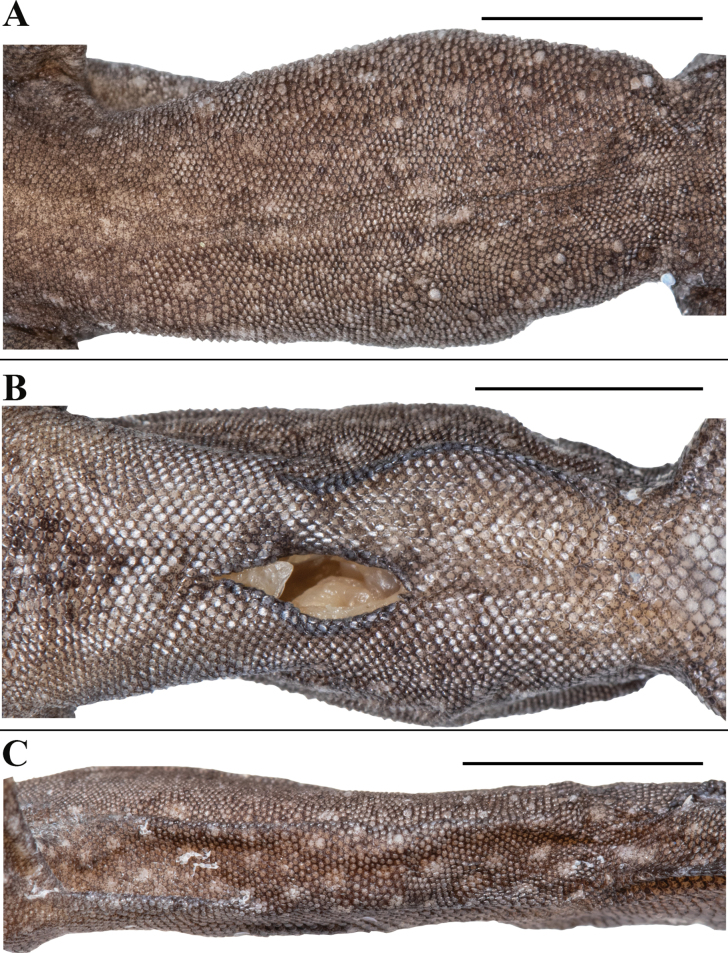
*Cnemaspissathuragiriensis* sp. nov. (holotype, NRC-AA-8349) **A** dorsal view of midbody **B** ventral view of midbody **C** lateral view of midbody. Photos by Akshay Khandekar. Scale bars: 5 mm.

**Figure 11.**
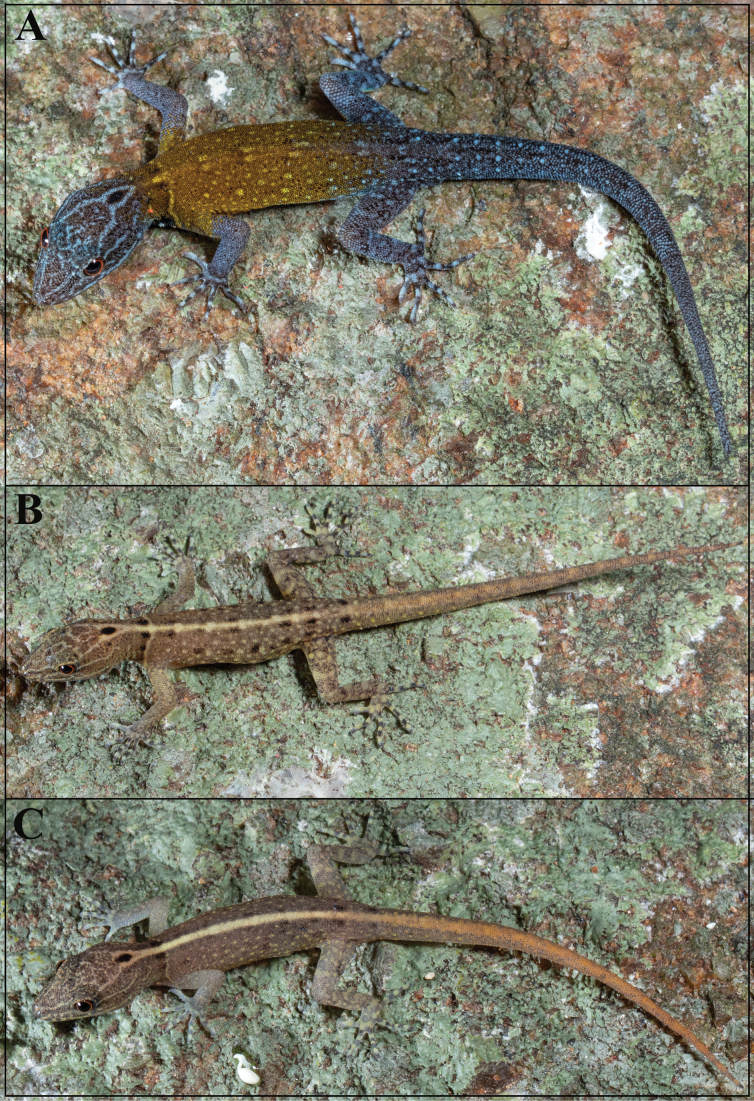
*Cnemaspissathuragiriensis* sp. nov., in life **A** adult male (holotype, NRC-AA-8349) **B** subadult female (paratype, NRC-AA-8353), and **C** juvenile (uncollected). Photos by Akshay Khandekar.

**Figure 12.**
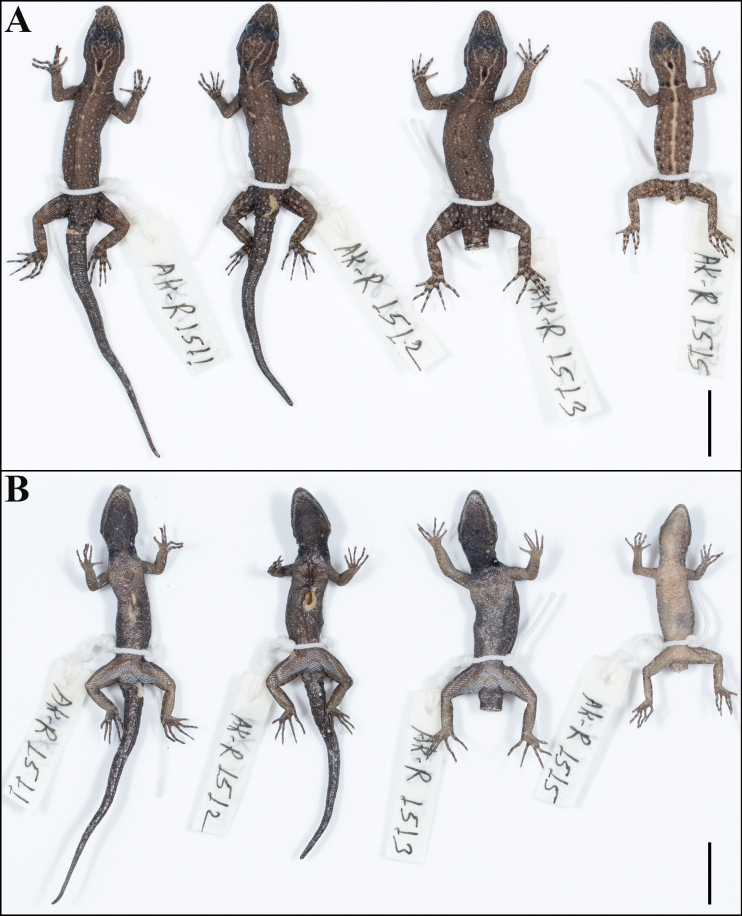
Paratype series of *Cnemaspissathuragiriensis* sp. nov., from left to right, NRC-AA-8350–8353 **A** dorsal view, and **B** ventral view. Photos by Akshay Khandekar. Scale bars: 10 mm.

Figs 8 , 9 , 10 , 11 , 12 , Tables 3 , 4 , 5

##### Type material examined.

***Holotype.***NRC-AA-8349 (AK-R 1510), adult male, from near Sathuragiri entry point (9.7093°N, 77.6307°E; ca 250 m a.s.l.), Sathuragiri, Virudhunagar district, Tamil Nadu state, India; collected by Akshay Khandekar, Ishan Agarwal, Swapnil Pawar and team on 26 April 2022. ***Paratypes.***NRC-AA-8350 (AK-R 1511), NRC-AA-8351 (AK-R 1512), adult males, same data as holotype; NRC-AA-8352 (AK-R 1513), adult male, NRC-AA-8353 (AK-R 1515), subadult female, from near Vazhukkuparai Saptur Reserve Forest (9.7174°N, 77.6244°E; ca 400 m a.s.l.), Sathuragiri; same data as holotype.

##### Diagnosis.

A small-sized *Cnemaspis*, snout to vent length ≤ 33 mm (*n* = 5). Dorsal pholidosis heterogeneous; smooth to weakly keeled granular scales intermixed with irregularly arranged rows of enlarged, weakly keeled, conical tubercles; 6–8 rows of dorsal tubercles at midbody, paravertebral tubercles either absent or irregular; ventral scales subequal from chest to vent, smooth, subcircular and subimbricate with rounded end; 28–30 midventral scales across belly, 130–137 longitudinal ventral scales from mental to cloaca; subdigital scansors smooth, unnotched, some divided and others entire, a distinct enlarged metacarpal scale below digit I; 11–13 lamellae under digit I of manus and 11 or 12 under digit I of pes, 18–21 lamellae under digit IV of manus and 23 or 24 lamellae under digit IV of pes; males with continuous series of seven or eight precloacal pores (*n* = 4); scales on non-regenerated tail dorsum heterogeneous; small, smooth, subcircular, flattened, subimbricate scales intermixed on anterior one third portion with enlarged, weakly keeled, and weakly conical tubercles forming eight whorls; six tubercles on first whorl, four tubercles on second to fourth whorls, only a pair of paravertebral tubercles each on fifth to eighth whorls; rest of the tail lacking enlarged tubercles; median row of subcaudals smooth, roughly subcircular, distinctly enlarged than rest, with condition of two enlarged scales alternating with a divided scale. Males with ochre dorsum, single central black dorsal ocellus on neck, a white ocellus on ventrolateral side of neck and one on throat posterior to jaw, venter off-white with dark throat, tail unbanded, females and juveniles brown with a prominent mid-dorsal streak.

##### Comparisons with members of *beddomei* clade.

*Cnemaspissathuragiriensis* sp. nov. can be easily distinguished from all 16 members of the *beddomei* clade as well as from *C.boiei* by a combination of the following differing or non-overlapping characters: A small-sized *Cnemaspis*, snout to vent length ≤ 33 mm (vs medium-sized *Cnemaspis*, snout to vent length 40–49 mm in *C.nairi*, *C.nimbus*, *C.ornata*, *C.rashidi*, *C.rubraoculus* and *C.wallaceii*; large-sized *Cnemaspis*, snout to vent length > 50 mm in *C.anamudiensis*, *C.beddomei*, *C.maculicollis*, and *C.smaug*; snout to vent length ≤ 38 mm in *C.azhagu*, *C.boiei*, and *C.nigriventris*); 6–8 rows of dorsal tubercles at midbody (vs only a few enlarged scattered tubercles at midbody dorsum in *C.anamudiensis*, two or three rows of dorsal tubercles at midbody in *C.azhagu*, 16–18 in *C.nairi*, 13 or 14 in *C.nigriventris*, 12–14 in *C.nimbus* and *C.ornata*, 19–22 in *C.smaug*, 10 in *C.vangoghi* sp. nov., 14 or 15 in *C.wallaceii*); 130–137 longitudinal ventral scales from mental to cloaca (vs 151–171 longitudinal ventral scales from mental to cloaca in *C.azhagu*, 154–161 in *C.beddomei*, 153–159 in *C.galaxia*, 143–147 in *C.nairi*, 154–159 in *C.nigriventris*, 157–165 in *C.ornata*, 170–172 in *C.rashidi*, 148–154 in *C.regalis*, 142–150 in *C.smaug*, 156–160 in *C.sundara*, 154–156 in *C.wallaceii*); paravertebral tubercles either absent or irregular (vs 18 or 19 tubercles in paravertebral rows in *C.aaronbaueri* and *C.beddomei*, 16 or 17 in *C.nimbus*, 21–23 in *C.ornata*, 27–30 in *C.smaug*, 7–14 in *C.vangoghi* sp. nov., 18–20 in *C.wallaceii*); 28–30 midventral scales across belly (vs 34–44 midventral scales across belly in *C.azhagu*, 32 or 33 in *C.nairi*, 38–40 in *C.nigriventris*, 26 or 27 in *C.nimbus*, 40–44 in *C.regalis*, 33–37 in *C.rubraoculus*, 35 or 36 in *C.sundara*); a distinct white ocellus on ventrolateral sides of neck present in males (vs white ocellus on ventrolateral sides of neck absent in *C.aaronbaueri*, *C.anamudiensis*, *C.azhagu*, *C.beddomei*, *C.maculicollis*, *C.nimbus*, *C.regalis*, *C.rubraoculus*, *C.smaug*, *C.wallaceii*); tail unbanded (tail distinctly banded in *C.nairi*, *C.nigriventris*, *C.ornata*, *C.rashidi*, *C.smaug*, *C.sundara*).

##### Description of the holotype.

Adult male in good state of preservation except tail marginally bent towards right, hemipenis fully everted on right, and a 4.1 mm long incision in sternal region for tissue collection (Fig. 8A–E); SVL 32.1 mm, head short (HL/SVL 0.27), wide (HW/HL 0.74), not strongly depressed (HD/HL 0.52), distinct from neck. Loreal region marginally inflated, canthus rostralis indistinct. Snout almost 1/2 of head length (ES/HL 0.44), 2× eye diameter (ES/ED 2.2); scales on snout and canthus rostralis subcircular to elongate, subequal, smooth, weakly conical, much larger than those on forehead and interorbital region; scales on forehead similar to those on snout and canthus rostralis except almost 2× smaller and elongate; scales on interorbital region, occipital, and temporal region even smaller, granular (Fig. 9A). Eye small (ED/HL 0.20); with round pupil; supraciliaries short, larger anteriorly; seven interorbital scale rows across narrowest point of frontal bone; 29 scale rows between left and right supraciliaries at mid-orbit level (Fig. 9A, C). Ear-opening deep, oval, small (EL/HL 0.05); eye to ear distance much greater than diameter of eye (EE/ED 1.61) (Fig. 9C). Rostral slightly > 2× as wide (1.7 mm) as high (0.8 mm), incompletely divided dorsally by a strongly developed rostral groove for > 1/2 of its height; a single enlarged, roughly rectangular supranasal on each side, almost 3× larger than upper postnasal, and strongly in contact with each other on snout; a pair of enlarged scales on snout behind internasals, separated from each other by a single smaller, granular scale; rostral in contact with supralabial I, nostril, and supranasal on either side; nostrils oval, surrounded by three postnasals, supranasal, rostral and supralabial I on either side; three postnasals on either side, the one touching supranasal largest, roughly rectangular, gradually decreasing in side posteriorly; two single row of scales separate orbit from supralabials (Fig. 9C). Mental enlarged, subtriangular, marginally wider (2.1 mm) than high (1.9 mm); two pairs of postmentals, inner pair roughly rectangular, shorter (1.0 mm) than mental, separated from each other below mental by a single enlarged median chin shield; inner pair bordered by mental, infralabial I, outer postmental, median chin shield and a single enlarged chin shields on either side; outer postmentals roughly rectangular, slightly smaller (0.6 mm) than inner pair, bordered by inner postmentals, infralabial I and II, and five enlarged chin shields on either side; three enlarged gular scales (including median chin shield) between left and right outer postmentals; all chin scales bordering postmentals more or less flattened, subequal, subcircular, smooth, and smaller than outermost postmentals; scales on rest of throat granular, smooth, subcircular, and juxtaposed scales (Fig. 9B). Infralabials bordered below by a row or two of slightly enlarged, much elongated scales, decreasing in size posteriorly. Eight supralabials up to angle of jaw and five at midorbital position on each side; supralabial I largest, gradually decreasing in size posteriorly; seven infralabials up to angle of jaw and five at midorbital position on either side; infralabial I largest, gradually decreasing in size posteriorly (Fig. 9C).

Body relatively slender (BW/AGL 0.50), trunk < 1/2 of SVL (AGL/SVL 0.39) without spine-like tubercles on flank (Fig. 10A–C). Dorsal pholidosis heterogeneous; smooth to weakly keeled granular scales intermixed with irregularly arranged rows of enlarged, weakly keeled, conical tubercles; granular scales gradually increasing in size towards each flank, largest on mid-flank; granular scales on occiput and nape slightly smaller than paravertebral granules; enlarged tubercles in approximately six longitudinal rows at midbody; enlarged tubercles in paravertebral rows absent, (Fig. 10A, C). Ventral scales much larger than granular scales on dorsum, subequal from chest to vent, smooth, subcircular and subimbricate with rounded end; scales on precloacal region and four or five rows on femur distinctly enlarged; midventral scale rows across belly 30; 132 ventral scales from mental to anterior border of cloaca (Fig. 10B). A continuous series of seven precloacal pores, femoral pores absent (Fig. 9D).

Scales on palm and soles granular, smooth, rounded, and flattened, a distinct enlarged metacarpal scale on palm below digit I; scales on dorsal aspects of limbs heterogeneous in shape and size; scales on upper arm and thigh much larger than granular scales on body dorsum, smooth and a few feebly keeled, slightly elongate, subimbricate with weakly pointed ends; scales on lower arm and shank granular, similar in size to granular scales on body dorsum, smooth, rounded, gradually becoming larger, flattened and subimbricate anterolaterally and posteriorly, largest on anterolateral aspect of the hands and feet; scales on ventral aspect of upper arm smooth, granular, much smaller than granular scales on body dorsum, scales on ventral aspect of lower arm with much larger scales than those on upper arm, smooth, subcircular and flattened scales; ventral aspect of thigh and shank with enlarged, smooth, flattened, subimbricate scales, much larger than midventrals (Fig. 8A, B). Forelimbs and hindlimbs slightly long, slender (LAL/ SVL 0.14); (CL/SVL 0.19); digits long, with a strong, recurved claw, distinctly inflected, distal portions laterally compressed conspicuously. Digits with both paired and unpaired lamellae, separated into a basal and narrower distal series by single enlarged lamella at inflection; one or two most basal paired on basal series and 1–4 paired lamellae above the inflection; basal lamellae series: (1-5-4-6-3 right manus, 1-6-7-8-2 right pes), (1-5-4-6-4 left manus, Fig. 9E; 1–6–7–8–2 left pes, Fig. 9F); distal lamellae series: (10-12-14-14-12 right manus, 10-12-16-16-16 right pes), (10-12-15-14-12 left manus, Fig. 9E; 10–13–16–15–15 left pes, Fig. 9F). Relative length of digits (measurements in mm in parentheses): IV (3.4) = III (3.4) > II (3.1) > V (3.0) > I (2.2) (left manus); IV (4.1) = III (4.1) > II (3.4) = II (3.3) > I (2.2) (left pes).

Tail mostly original with regenerated tip, entire, subcylindrical, slender, marginally longer than body (TL/SVL = 1.14) (Fig. 8C–E). Dorsal pholidosis on tail heterogeneous; small, smooth, subcircular, flattened, subimbricate scales intermixed on anterior one third portion with enlarged, weakly keeled, and weakly conical tubercles forming eight whorls; six tubercles on first whorl, four tubercles on second to fourth whorls, only a pair of paravertebral tubercles each on fifth to eighth whorls; rest of the original and regenerated tail lacking enlarged tubercles (Fig. 8C, E). Scales on tail venter much larger than those on dorsal aspect, smooth, roughly subcircular, flattened, subimbricate; median series smooth, roughly subcircular, distinctly enlarged than rest, with condition of two enlarged scales alternating with a divided scale (Fig. 8D). Scales on tail base much smaller, smooth, imbricate; a single enlarged, smooth, and weakly conical postcloacal tubercle on each side (Fig. 8D, E).

##### Colouration in life

**(Fig. 11A).** Dorsal ground colour of body, limbs, and tail pale grey; neck and trunk ochre, fading slightly near hindlimb insertions. Pale blue-grey preorbital streak runs from nostril to orbit; three pale postorbital streaks, uppermost on either side meeting in parietal region forming an inverted chevron enclosing a single large elongate black ocellus on occiput, middle terminating on neck and lowermost continuing until ear opening. Head finely reticulated with pale blue-grey, a white ocellus on a black patch of scales on each side of ventrolateral aspect of neck just anterior to forelimb insertions; a fine yellow collar at anterior edge of forelimb insertions, broken in the centre, two fine black spots anterior to and a yellow spot on the division. Fine black spots and paler blotches on dorsum, tubercles and a few adjacent scales around hindlimb insertions and on tail pale blue-grey; similar spots on posterior flank, femur and bands on tibia; upper 1/2 of upper arm ochre, otherwise whitish-grey with dark outlines of scales; digits with white and dark markings. Original tail without bands, blue with dark outlines of scales and darker markings. Ventral ground colouration grey-white; throat fairly strongly marked with black up to forelimb insertions except for a fine pale border just below infralabials, a white spot on either side of the throat posterior to jaw; belly with scattered dark markings and blue-grey scales toward the lateral margins; underside of limbs and tail with few dark markings; precloacal and femoral region with almost no dark markings. Pupil black, iris dark red with a pale orange ring lining pupil.

##### Variation and additional information from type series

**(Figs 11B, C, 12).** Mensural, meristic, and additional character state data for the type series is given in Tables 3–5, respectively. There are three adult males, and a single subadult female ranging in size from 26.7–33.0 mm (Fig. 12). All paratypes resemble the holotype except as follows: inner postmentals bordered by mental, infralabial I, outer postmental, enlarged median chin shield in all paratypes, additionally, bordered by two small chin scales on left and a single scale on right side in NRC-AA-8351. Outer postmentals bordered by inner pair, infralabial I & II in all paratypes, additionally, bordered by four chin scales on left and five on right side in NRC-AA-8350, four on left and five on right side in NRC-AA-8351 and NRC-AA-8353, four on either side in NRC-AA-8352; outer postmental separated from each other by four chin scales including median chin shield in NRC-AA-8351. NRC-AA-8350 with almost original tail with tip regenerated, marginally longer than body (TL/SVL 1.17), NRC-AA-8351 with original tail with missing tail tip, equal to body (TL/SVL 1.02); Two paratypes, NRC-AA-8352 and NRC-AA-8353 with completely missing tails; NRC-AA-8351 with damaged skink on the tail base; NRC-AA-8350 with fully everted hemipenis only on left side.

The new species is strongly sexually dichromatic and shows ontogenetic colour variation (Fig. 11A–C): subadult female pale brown with a cream mid-dorsal streak that continues onto tail formed by the extension of the neck chevron, dorsum with scattered black and pale blotches, collar pale brown, flanked anteriorly by a few black spots; distinct black central ocellus on occiput, white ocelli on side of neck absent; forelimbs brown, hindlimbs with scattered dark and pale markings, digits banded. Original tail grey, without bands, regenerated portion blue in male paratypes. Ventral ground colouration of gular, body and tail grey-white; underside of limbs with few dark markings. Juveniles brown with a cream mid-dorsal streak that continues onto tail where it is orange formed by the extension of the neck chevron; distinct black central ocellus on occiput, white ocelli on side of neck absent; forelimbs brown, hindlimbs with scattered dark and pale markings, digits banded. Original tail grey, without bands, regenerated portion blue in male paratypes (Fig. 12A, B). Ventral ground colouration of gular, body and tail grey-white; underside of limbs with few dark markings.

##### Etymology.

The specific epithet is a toponym for the type locality of the new species, Sathuragiri mountain in Srivilliputhur-Megamalai Tiger Reserve (SMTR), Virudhunagar District, Tamil Nadu. Suggested Common name is Sathuragiri dwarf gecko.

##### Distribution and natural history.

**Figure 13.**
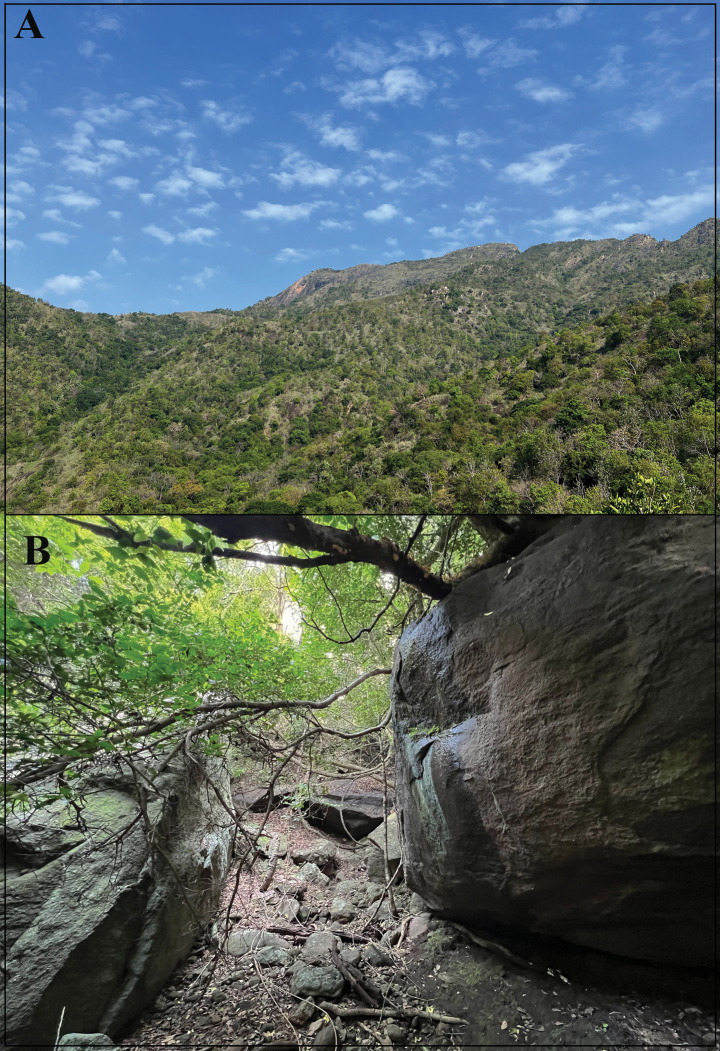
Habitat of *Cnemaspissathuragiriensis* sp. nov. at the type locality **A** general view, and **B** microhabitat from where types were collected. Photos by Akshay Khandekar.

*Cnemaspissathuragiriensis* sp. nov. is known only from its type locality (Sathuragiri hills in Virudhunagar district, Tamil Nadu) between elevations of 250–400 m a.s.l. on eastern slopes of the Western Ghats (Fig. 1). Individuals of the new species were observed active during the daytime (0830–1100 hrs) on rocks and tree trunks < 2 m high from the base in seasonally dry tropical forest with a mix of evergreen and deciduous species (Fig. 13A). The species was observed in high abundance (*n* ≥ 20/hr), more commonly on boulders in shaded areas than tree trunks (Fig. 13B). Sympatric lizards at the type locality include Cnemaspiscf.gracilis, Hemidactyluscf.frenatus, H.cf.leschenaultii, *H.vanam*, *Dravidosepssrivilliputhurensis*, *Eutropiscarinata*, *E.macularia*, and Psammophiluscf.blanfordanus.

### ﻿Key to the *ornata* subclade within the *beddomei* clade of South Asian *Cnemaspis*

**Table:** 

1	White ocellus on ventrolateral sides of neck in males	**2**
–	No white ocellus on ventrolateral sides of neck in males	**7**
2	Original tail unbanded	**3**
–	Original tail banded	**5**
3	≤ 140 longitudinal scales from mental to cloaca	**4**
–	153–159 longitudinal scales from mental to cloaca	***C.galaxia***
4	Entire dorsum ochre in adult males	***C.sathuragiriensis* sp. nov.**
–	Anterior 1/2 of dorsum ochre in adult males	***C.vangoghi* sp. nov.**
5	150–165 longitudinal scales from mental to cloaca	**6**
–	143–147 longitudinal scales from mental to cloaca	***C.nairi***
–	170–172 longitudinal scales from mental to cloaca	***C.rashidi***
6	Paravertebral tubercles absent	***C.sundara***
–	15 or 16 paravertebral tubercles	***C.nigriventris***
–	21–23 paravertebral tubercles	***C.ornata***
7	Paravertebral tubercles irregular or absent	***C.azhagu***
–	18 or 19 paravertebral tubercles	***C.aaronbaueri***
–	15 or 16 paravertebral tubercles	***C.regalis***

## ﻿Discussion

The *ornata* subclade now has 11 known valid species (including the two new species described in this paper) in a small geographic area spanning < 1° longitude and 1.5° latitude. At the southern extreme of the Western Ghats, the region is incredibly heterogeneous, with altitudinal variation from close to sea level to > 1,500 m a.s.l. and strong east-west gradients in total annual precipitation and seasonality. Habitats range from thorny scrub forest on the lower eastern slopes of the mountains to evergreen forest at higher elevations and on the western slopes. This subclade is distributed across the Shencottah Gap (SG), a relatively low elevation pass through the Western Ghats. All 11 members of the clade are strongly sexually dichromatic, and sexual selection may at least in part be a driver of the high diversity in this clade, as has been speculated for members of the *C.gracilis* clade (Agarwal et al. 2022). The two new species add to the five previously known endemic vertebrates from Srivilliputhur-Megamalai Tiger Reserve – the geckos *Cnemaspisgalaxia*, *C.rashidi*, *Hemidactylusvanam*; the skink *Dravidosepssrivilliputhurensis* and the anuran *Nasikabatrachusbhupathyi* Janani, Vasudevan, Prendini, Dutta & Aggarwal, 2017 (Janani et al. 2017; Chaitanya et al. 2018; Pal et al. 2021; Sayyed et al. 2023a, b; Agarwal et al. 2024).

Though sampling of the *ornata* subclade likely remains incomplete as this vast mountainous landscape has a number of higher elevations we could not access in our rapid surveys, there are some geographic patterns that emerge based on available data. The only two high elevation species are the sister pair *C.ornata* + *C.rashidi* that are distributed north of the SG, together forming the sister taxon to the low elevation species *C.nairi* + *C.nigriventris* that are distributed around the SG. These two sister pairs are deeply divergent from one another, indicative of potentially more undiscovered species in the intervening areas. The subclade containing *C.aaronbaueri*, *C.azhagu* and *C.regalis* is distributed entirely south of the SG and mainly on the eastern slopes, while the final subclade includes the low to mid elevation *C.sundara* which is distributed close to the SG, and the *galaxia* complex with three low elevation species in Srivilliputhur. Large sampling gaps exists between the distribution of *C.sundara* and *C.galaxia* as well as between *C.nairi* and *C.regalis*. Pal et al. (2021) considered the pairs *C.galaxia* + *C.regalis* and *C.nairi* + *C.nigriventris* to be separated by the Shencottah Gap — although we now know that each member of the former pair represents a cluster of closely related species, and at least *C.nigriventris* spans the Shencottah Gap. It is unclear what the northern boundary of the *ornata* subclade is, and we sampled areas north of Srivilliputhur including the Anaimalai and Palani Hills but failed to locate any species of the *ornata* subclade.

This last section is a note on violations of Principle 2 of the Code of Ethics prescribed by The Code (appendix A; Anonymous 1999) which states

“*A zoologist should not publish a new name if he or she has reason to believe that another person has already recognized the same taxon and intends to establish a name for it (or that the taxon is to be named in a posthumous work). A zoologist in such a position should communicate with the other person (or their representatives) and only feel free to establish a new name if that person has failed to do so in a reasonable period (not less than a year).*”

One of the authors of *Cnemaspisrashidi* accompanied us in the field in 2022 when we collected the then unnamed and distinctively coloured species, and multiple co-authors including the first author were aware that we were working in Tamil Nadu on *Cnemaspis* among other lizards (Sayyed et al. 2023a, b). While it is not unexpected that multiple workers may find the same undescribed species, what happens next is important. This is a matter of concern for the scientific community at large, and the Indian herpetological community in particular. In two other cases, even after we (AK, IA) explicitly initiated discussions with two groups whom we knew had collected the same species we were in the process of describing, the other teams went ahead with their descriptions without consultation, of *Eublepharispictus* Mirza & Gnaneswar, 2022 and Cyrtodactylus (Geckoella) aravindiNarayanan et al., 2022 (Mirza and Gnaneswar 2022; Narayanan et al. 2022). While there are more than enough species to go around, it is in contravention of the Code of Ethics, besides being a waste of time, effort, and resources when teams compete against one another instead of coming together to collaborate and increase the amount of data available in a species description.

## Supplementary Material

XML Treatment for Cnemaspis vangoghi

XML Treatment for Cnemaspis sathuragiriensis

## ﻿Additional information

### Author ORCIDs

Akshay Khandekar https://orcid.org/0000-0002-7956-089X

Tejas Thackeray https://orcid.org/0000-0002-9981-8763

Ishan Agarwal https://orcid.org/0000-0001-9734-5379

### Data availability

All of the data that support the findings of this study are available in the main text.

## ﻿Appendix 1

**Material examined.**

Institutional and field series abbreviations are as follows: National Centre for Biological Sciences, Bengaluru (NCBS-AU/ NCBS-BH/NRC-AA); Bombay Natural History Society, Mumbai (BNHS); Zoological Survey of India, Western Ghats regional station Kozhikode (ZSI/WGRC), and Akshay Khandekar field series (AK-R).

*Cnemaspisazhagu*: holotype, NRC-AA-1170 (adult male); paratypes, NRC-AA-1171, NRC-AA-1172, NRC-AA-1174, BNHS 2818, BNHS 2819, BNHS 2820 (adult males), BNHS 2821, (adult female), NRC-AA-1173 (subadult female), from Thirukurungudi forest range, Kalakad Mundanthurai Tiger Reserve, Tirunelveli District, Tamil Nadu state, India.

*Cnemaspisbeddomei*: AK-R 578, AK-R 579, AK-R 581–583 (adult males), AK-R 580 (adult female), from Kakachi, Kalakkad-Mundanthurai Tiger Reserve, Tirunelveli District, India.

*Cnemaspisgalaxia*: holotype, BNHS 2626 (adult male), from low elevation riparian forest, Srivilliputhur, Tamil Nadu; AK-R 1422, AK-R 1323, AK-R 1324, AK-R 1428, AK-R 1470, AK-R 1471, AK-R 1473 (all adults) from near Shenbaga Thopu, Srivilliputhur, Virudhunagar District, Tamil Nadu, India.

*Cnemaspisnairi*: AK-R 894, AK-R 895 (adults), from Thenmala, Courtallam, Tenkasi District, Tamil Nadu, India.

*Cnemaspisnigriventris*: holotype, BNHS 2619 (adult male), from Achankovil Reserve Forest, Kollam District, Kerala; AK-R 1265 and AK-R 1275 (adults), from Mohan’s resort, Tenkasi District, Tamil Nadu, India.

*Cnemaspisnimbus*: holotype, BNHS 2614 (adult male), from Mathikettan Shola National Park, Cardamom Hills, Idukki District, Kerala, India.

*Cnemaspisrashidi*: AK-R 1439 and AK-R 1440 (adults), from high elevation of Shenbaga Thopu; AK-R 1692 and AK-R 1693, from Vellimalai, Srivilliputhur-Megamalai Tiger Reserve, Virudhunagar and Theni Districts, Tamil Nadu, India.

*Cnemaspisregalis*: holotype, BNHS 2617 (adult male); AK-R 453, AK-R 454, AK-R 453 (adults), from Mundanthurai, Kalakkad-Mundanthurai Tiger Reserve, Tirunelveli District, Tamil Nadu, India.

*Cnemaspisrubraoculus*: holotype, BNHS 2612 (adult male) from Upper Manalar, Periyar Tiger Reserve, Megamalai; AK-R 1728, AK-R 1729 (adults), from Vellimalai, AK-R 1777 (adults), from Megamalai, Srivilliputhur-Megamalai Tiger Reserve, Theni District, Tamil Nadu, India.

*Cnemaspissmaug*: holotype, BNHS 2615 (adult male) from Mathikettan Shola National Park, Cardamom Hills, Idukki District, Kerala, India.

*Cnemaspissundara*: AK-R 1254 and AK-R 1266 (adults), from Mohan’s resort, Tenkasi District, Tamil Nadu, India.

*Cnemaspiswallaceii*: holotype, BNHS 2613 (adult male), from Andiparai Shola, Anamalai Hills, Coimbatore District, Tamil Nadu, India.
